# Earth system feedback statistically extracted from the Indian Ocean deep-sea sediments recording Eocene hyperthermals

**DOI:** 10.1038/s41598-017-11470-z

**Published:** 2017-09-12

**Authors:** Kazutaka Yasukawa, Kentaro Nakamura, Koichiro Fujinaga, Minoru Ikehara, Yasuhiro Kato

**Affiliations:** 1 Department of Systems Innovation, School of Engineering, The University of Tokyo, 7-3-1 Hongo, Bunkyo-ku, Tokyo 113-8656 Japan; 2 Ocean Resources Research Center for Next Generation, Chiba Institute of Technology, 2-17-1 Tsudanuma, Narashino, Chiba 275-0016 Japan; 3 Frontier Research Center for Energy and Resources, School of Engineering, The University of Tokyo, 7-3-1 Hongo, Bunkyo-ku, Tokyo 113-8656 Japan; 4 Center for Advanced Marine Core Research, Kochi University, B200 Monobe, Nankoku, Kochi 783-8502 Japan; 5 Research and Development Center for Submarine Resources, Japan Agency for Marine-Earth Science and Technology (JAMSTEC), 2-15 Natsushima-cho, Yokosuka, Kanagawa 237-0061 Japan

## Abstract

Multiple transient global warming events occurred during the early Palaeogene. Although these events, called hyperthermals, have been reported from around the globe, geologic records for the Indian Ocean are limited. In addition, the recovery processes from relatively modest hyperthermals are less constrained than those from the severest and well-studied hothouse called the Palaeocene–Eocene Thermal Maximum. In this study, we constructed a new and high-resolution geochemical dataset of deep-sea sediments clearly recording multiple Eocene hyperthermals in the Indian Ocean. We then statistically analysed the high-dimensional data matrix and extracted independent components corresponding to the biogeochemical responses to the hyperthermals. The productivity feedback commonly controls and efficiently sequesters the excess carbon in the recovery phases of the hyperthermals via an enhanced biological pump, regardless of the magnitude of the events. Meanwhile, this negative feedback is independent of nannoplankton assemblage changes generally recognised in relatively large environmental perturbations.

## Introduction

**Figure 1:**
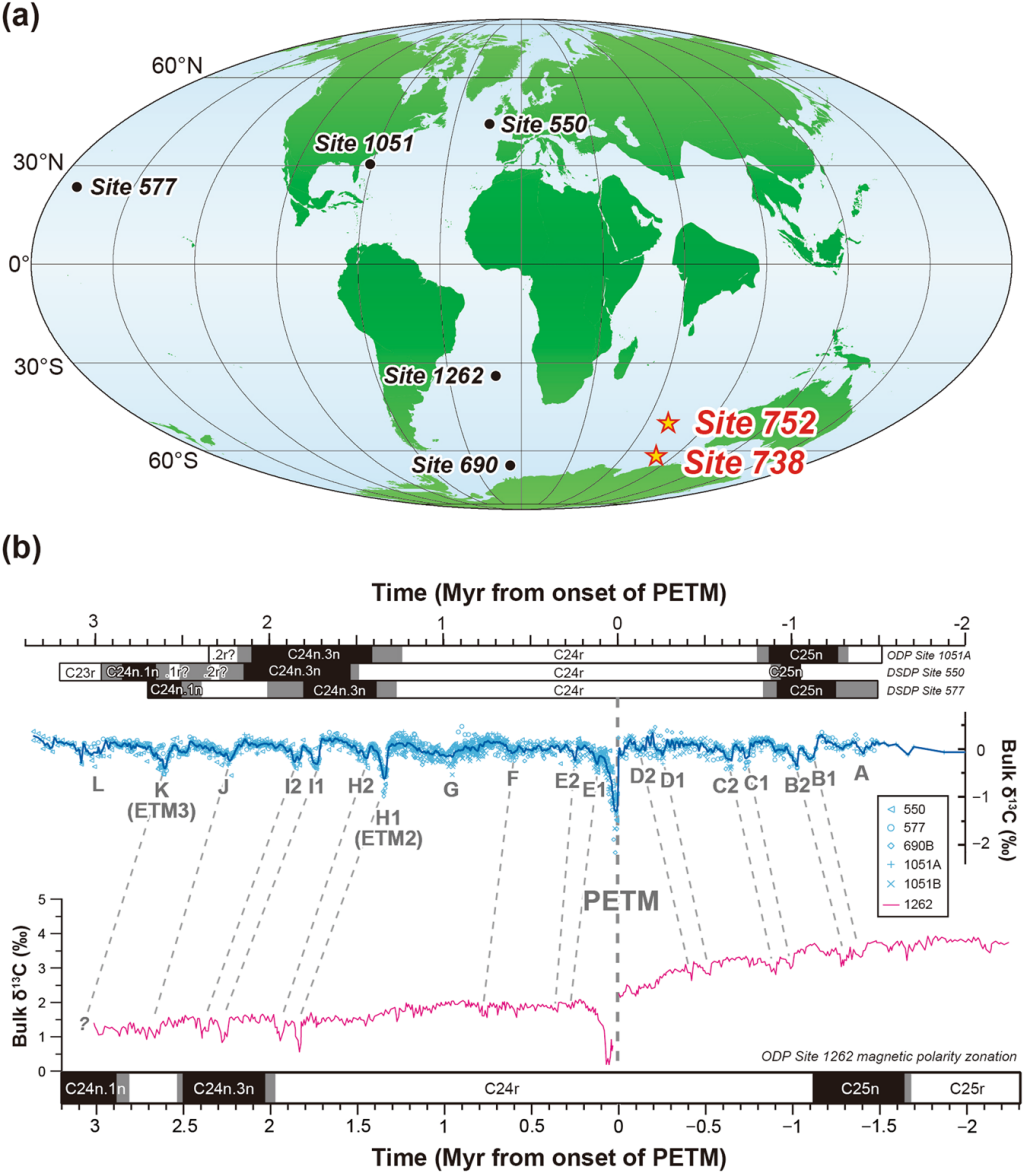
Palaeogeographic map at 55 Ma and compilation of bulk carbon isotopic (δ^13^C) records of the Palaeocene–Eocene deep-sea sediments. (**a**) Approximate palaeolocations of the sites newly analysed in this study (yellow stars). The circles filled in black indicate the sites providing the published data stacks in panel (**b**). The map was created using the GPlates software (http://www.gplates.org) version 1.2.0 with the EarthByte Global Rotation Model^62^ and the Global Time-Dependent Age Grid^63^, and Generic Mapping Tools software (https://www.soest.hawaii.edu/gmt/), version 4.5.8^64^. (**b**) High-resolution bulk δ^13^C records from Deep Sea Drilling Project/ODP cores in the Atlantic and Pacific Oceans obtained in two previous studies^16, 28^. The data plotted in blue are detrended in the original study^16^, whereas the magenta line reflects the raw data^28^. The alphabets partly with numbers represent CIEs corresponding to each hyperthermal event, which were assigned by Cramer *et al*.^16^. Although both δ^13^C time series are plotted in relative ages with respect to the onset of the PETM, the age models between the two previous reconstructions differ. The magnetic polarity zonations are from each of the original papers^16, 28^.

During the early Palaeogene, ~59 to 50 million years (Ma) ago, Earth’s mean surface temperature and the partial pressure of the atmospheric CO_2_ were considerably higher than at present^1^. Superimposed on the long-term global warming trend, one of the most prominent environmental perturbations of the last 65 Ma, the Palaeocene–Eocene Thermal Maximum (PETM), occurred at ~56 Ma^1, 2^. Three key features characterise the PETM: rapid (within several thousand years) and extreme (by 5 °C to 8 °C) global warming; distinct negative carbon isotope (δ^13^C) excursions (CIEs; Fig. 1b), in both marine and terrestrial realms; and severe ocean acidification resulting in widespread deep-sea carbonate dissolution. The global co-occurrence of these incidents strongly suggests that a rapid and tremendous injection of ^13^C-depleted greenhouse gas into the ocean–atmosphere system, that is to say, thousands of petagrams of CO_2_ and/or CH_4_^3–9^, caused the dramatic perturbation of the Earth’s surface (i.e. ocean, atmosphere, and biosphere), carbon cycle, and the climate. It has been suggested that negative feedback processes of the Earth system, such as the increase of silicate weathering and biological productivity, effectively controlled and sequestered the excess carbon during the recovery phase^10–15^.

Moreover, multiple PETM-like global warming episodes, termed ‘hyperthermals’, accompanying rapid and pronounced negative CIEs (Fig. 1) during the early Eocene period (~56–52 Ma), have been recognised over the past dozen years. Geologic hyperthermal records have been reported from around the globe such as from the Pacific^16, 17^, the Atlantic^16, 18, 19^, and the Arctic Oceans^20^ and Oceania^21^, Europe^22, 23^, and North America^24^. However, albeit its breadth and geographical importance to connect the Atlantic and Pacific Oceans, only a few published data are available on the Indian Ocean hyperthermals^25, 26^.

Another information gap exists between the PETM and other minor hyperthermals. The magnitude of the CIE and the degree of inferred warming have been generally proportional among the hyperthermals, including the PETM, suggesting that the isotopic composition and the release mechanism of the greenhouse gas responsible for these events were essentially similar and thus, the mass of injected carbon primarily controlled the magnitude of the perturbation of the global climate and carbon cycle^19, 24, 27^. A number of authors have suggested repetitive carbon release via oxidation of peat^28^ or melting of CH_4_ hydrate^27^ or permafrost^9^ controlled by Earth’s orbital forcing to be the possible causes of hyperthermals. However, in contrast to the PETM, the mechanisms for the recovery from multiple Eocene hyperthermals with relatively modest environmental perturbations still remain poorly constrained.

To fill these significant information gaps among Palaeogene warmings, we first present new and high-resolution geochemical records, including the PETM and multiple Eocene hyperthermals, from sediment cores at two sites in the Indian Ocean obtained by the Ocean Drilling Program (ODP). We demonstrate that the sediments of these cores clearly record multiple Eocene hyperthermals, which confirms that environmental perturbations less severe than the PETM were certainly global phenomena. Next, we employ a new multivariate statistical approach based on independent component analysis (ICA), which was originally established in the fields of neuro- and information- sciences^29^. By applying the ICA to the new dataset, we extract statistically independent geochemical signatures hidden in the high-dimensional data structure and characterise the biogeochemical processes during the series of transient perturbations in the Indian Ocean.

## Results

### Studied sites and stratigraphic constraints

**Figure 2:**
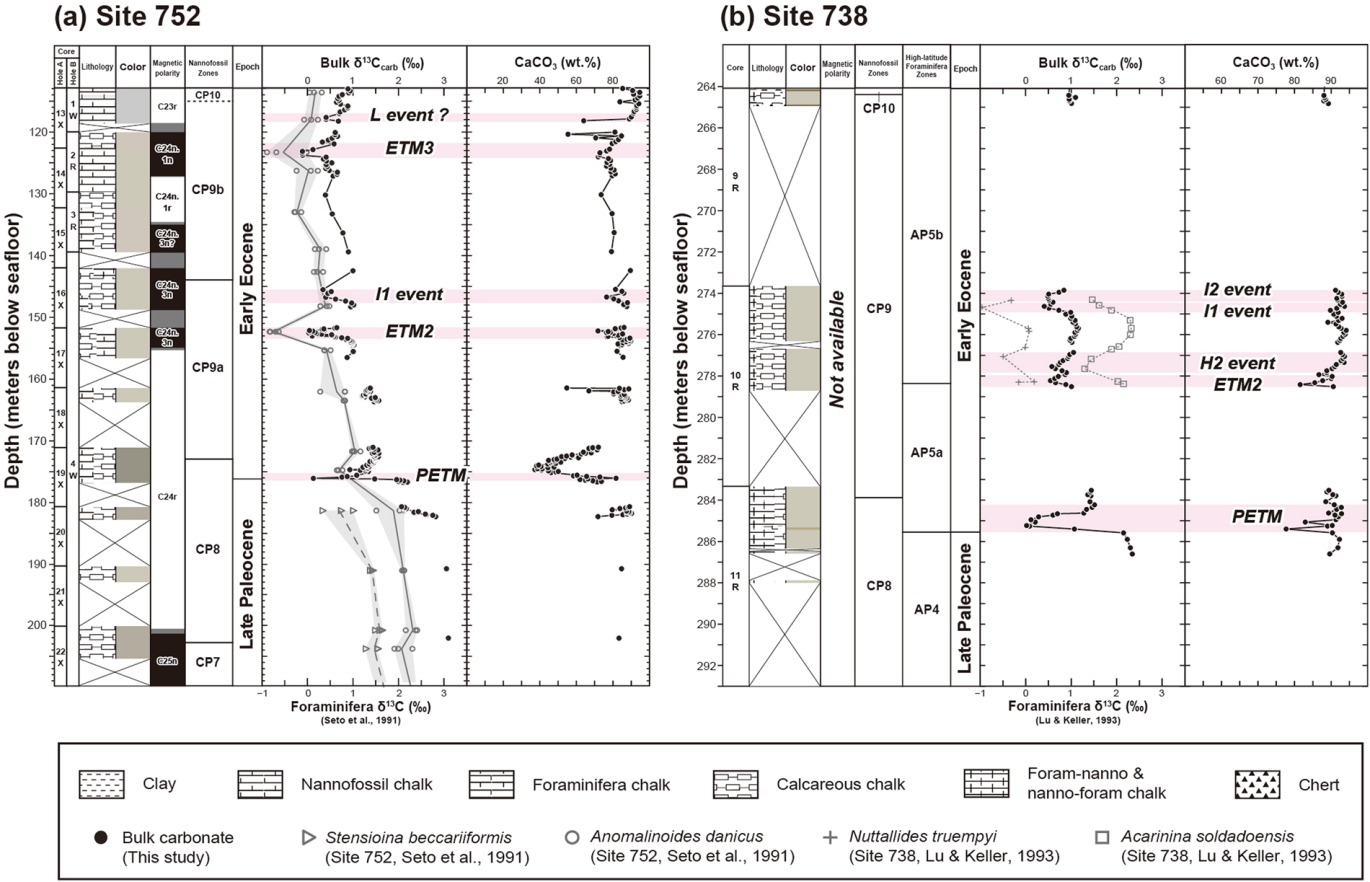
Data summary of the early Palaeogene sequences at two studied ODP sites. (**a**) Site 752 and (**b**) Site 738. The lithology, sediment colour, magnetic polarity, and nannfosossil zones are taken from the ODP Initial Reports of each site^30, 31^, whereas the carbon isotope ratios (δ^13^C_carb_) and CaCO_3_ contents of bulk carbonate were analysed in this study. The high-latitude foraminifera zones are based on ref. 35. The horizontal pink bars indicate the CIEs corresponding to the hyperthermal events. At Site 752, information and data from Holes 752A and 752B are integrated. The literature data using (**a**) two benthic foraminiferal species, *Stensioina beccariiformis* and *Anomalinoides danicus*, at Site 752^34^ and (**b**) benthic foraminifera, *Nuttallides truempyi*, and surface dwelling planktonic foraminifera, *Acarinina soldadoensis*, at Site 738^35^ are also plotted for comparison.

Deep-sea sediment samples were obtained from the ODP Sites 752 and 738 located in the southern Indian Ocean and the Indian sector of the Southern Ocean, respectively (Fig. 1a). The ODP Site 752 is located at 30°53.48′S, 93°34.65′E, and 1,086 m water depth, near the crest of Broken Ridge. Palaeogeographic reconstruction indicates that the palaeoposition of this site at ~55 Ma was 49°51′S and 70°46′E, close to the Palaeocene–Eocene boundary (Fig. 1a). The sediment at Site 752 mainly consists of nannofossil to calcareous chalk and partly contains volcanic ash resulting in a darker grey sediment colour^30^ (Fig. 2a). The ODP Site 738 is located at 62°42.54′S, 82°47.25′E, and 2,253 m water depth in the southern part of the Kerguelen–Heard Plateau. The palaeoposition of this site at ~55 Ma was 61°59′S and 79°13′E (Fig. 1a). The sediment at Site 738 consists of calcareous nannofossil ooze and chalk to calcareous chalk and partly contains chert nodules and fragments^31^ (Fig. 2b). In this study, we determined the bulk carbonate δ^13^C and CaCO_3_ content of the samples and integrated the new data with published compositional data^32^ (see Methods and Supplementary Data).

There are multiple coring gaps at both the sites (Fig. 2), resulting in missing continuous sedimentary records and less constrained stratigraphy than other well-investigated sites^16–20, 28^. Because only one hole was recovered across the Eocene hyperthermal intervals at each site, a composite depth scale based on the compilation of multiple complementary holes obtained with the recent more sophisticated drillings^28^, could not be established. In addition, the drilling was accomplished with extended or rotary core barrels, which produced a lot of coring disturbances^30, 31^. Notwithstanding these disadvantages, the available biostratigraphic and magnetostratigraphic data can provide chronological constraints on the apparent ‘stratigraphic mess’ in the ODP legacy cores^33^ (see Methods).

On the basis of the chronological information and stratigraphic relationships of the newly obtained isotopic signals, i.e. the negative CIEs, we can identify the signature of each hyperthermal event as described below. However, considering significant discontinuities in each core, we avoided assigning a numerical age to each sample horizon by a simple interpolation. Instead, we describe the results by using the original depth assigned by the ODP.

### Bulk sediment geochemical records

At Site 752, the bulk carbonate δ^13^C shows a distinct negative CIE of ~1.8‰ and subsequent recovery between 176.36 and 175.16 mbsf (Fig. 2a). Considering the amplitude of the CIE and relative position of the horizon in the biostratigraphy and magnetostratigraphy, this event corresponds to the PETM. After the exponential pattern of recovery, another small negative CIE of ~0.3‰ can be observed at 174.76 mbsf (Fig. 2a). This smaller CIE might be the E1 or E2 event reported by Cramer *et al*.^16^ (Fig. 1b). On the other hand, the CaCO_3_ content during the PETM does not show a coherent decrease. This indicates that the water depth at this site was sufficiently shallow as opposed to the lysocline and carbonate compensation depth (CCD), despite the CCD shoaling during the PETM^6–8^. Notably, the CaCO_3_ profile exhibits a broad negative peak that does not correspond to the δ^13^C profile between 177 and 171 mbsf (Fig. 2a). This reflects the relative decrease of the CaCO_3_ content due to an admixture of volcanic ash^30^.

Between 153.46 and 151.73 mbsf at Site 752, δ^13^C shows a negative CIE of ~0.9‰ (Fig. 2a). This CIE amplitude is comparable to other bulk carbonate records of the Eocene Thermal Maximum 2 (ETM2) from the Atlantic^16, 28^ and Pacific Oceans^16^ (Fig. 1b). In the magnetostratigraphy, this CIE is situated near the base of C24n.3n, which also supports this interpretation when compared with the other sites (Fig. 1b). While δ^13^C appears to show a single peak, except for one outlier, the CaCO_3_ content exhibits two negative peaks in this interval. Between 147.72 and 145.56 mbsf, another negative CIE of ~0.6‰ occurs within the normal chron C24n.3n. This CIE amplitude is similar to that of the I1 event (Fig. 1b; refs 16, 28). Subsequently, between 124.08 and 121.93 mbsf, a negative CIE of ~0.5‰ can be recognised (Fig. 2a). This interval is situated in the normal polarity just below the base of chron C23r (Fig. 2a), which indicates that the CIE corresponds to the ETM3 within C24n.1n^22, 23^ (Fig. 1b). Furthermore, the upper horizon between 118.24 and 117.00 mbsf also shows a smaller negative CIE (~0.3‰; Fig. 2a), which might be associated with the L event reported by Cramer *et al*.^16^ (Fig. 1b).

Although each horizon of hyperthermals exhibits a decrease in the CaCO_3_ content to various extents, the CaCO_3_ content generally does not decline below 70 wt.%, which indicates that the water depth at Site 752 had been shallower than the CCD throughout the early Eocene. The overall pattern of the bulk δ^13^C profile is consistent with that of benthic foraminiferal δ^13^C of a previous study^34^ (Fig. 2a). Although the previous work did not capture the CIE at the PETM due to coarse sampling, two distinct negative δ^13^C peaks derived from benthic foraminifera are clearly coincident with our detailed bulk δ^13^C data, indicating the ETM2 and ETM3.

At Site 738, the bulk carbonate δ^13^C shows a negative excursion of ~2.1‰ between 285.59 and 284.23 mbsf (Fig. 2b), which corresponds to the PETM horizon^35^. Higher-resolution bulk carbon isotopic data indicate that the full amplitude of the negative CIE was ~2.5‰^36^. Although the decrease of the CaCO_3_ content accompanies the CIE, even the lowest value is as high as 78 wt.% and the lithology remains carbonate throughout the interval investigated here. This means that the water depth at Site 738 was also sufficiently shallower than the CCD (palaeodepth: ~1,350 mbsf; ref. 35).

A pair of negative CIEs of ~0.5‰ can be recognised between 278.52 and 276.87 mbsf (Fig. 2b). In addition, another distinct negative CIE of ~0.6‰ occurs between 274.93 and 273.86 mbsf. Although less clear than the former ones, the latter one also appears to have twin peaks. The chemostratigraphic characteristic of the occurrence of successive twin peaks of negative CIEs strongly suggests that they correspond to a series of ETM2/H2 and I1/I2 events^16, 21, 28^ (Fig. 1b). This is consistent with the observation that all of these events occurred in the nannofossil zone NP11^16, 17, 21–23^ corresponding to the nannofossil zone CP9^25, 37^ (Fig. 2b). With respect to the onset of the ETM2 at Site 738, its sedimentary record could have been lost due to a core gap (Fig. 2b) and thus the true amplitude of the CIE might be larger (up to ~1‰; Fig. 1b). The general pattern of our bulk δ^13^C profile through this interval agrees with the well-preserved planktonic foraminiferal δ^13^C profile^35^ (Fig. 2b), which indicates that our bulk data from Site 738 certainly capture the carbon cycle perturbation during the early Eocene.

### Independent Component Analysis

To decipher complicated variations in the chemical composition of the bulk sediment due to an admixture of multiple source materials and/or processes, multivariate statistical analyses are likely to be very powerful tools because they comprehensively utilise multi-elemental information. However, such a short-term and prominent environmental perturbation can produce a variety of marginal or minor features deviating from the general trend in the entire data structure via unusual shifts, both in elemental and isotopic compositions. These marginal features result in a non-Gaussian data structure (e.g. highly skewed histograms of the variables deviating from an ‘analysis-friendly’ Gaussian distribution; Supplementary Fig. S1), which makes it unsuitable to apply some types of multivariate analytical techniques given the observed data constitute a multivariate Gaussian distribution^29^. Hence, instead of traditional methods, such as principal component analysis (PCA) or factor analysis, both intrinsically assuming the Gaussianity of the data, we employed ICA to identify independent geochemical signatures hidden in the high-dimensional data structure with clear non-Gaussianity.

In the present work, we applied ICA to the multi-elemental dataset of 249 bulk sediment samples. The ICA is a statistical method to transform observed multidimensional signals into a linear combination of base vectors with minimal statistical dependencies between each other. These new base vectors are called ‘independent components (ICs)’. The ICA is widely used for various types of signal processing such as blind source separation, denoising, and sparse coding^29^. Its utility has also been recognised in geochemistry; indeed, ICA provided successful results in previous studies dealing with large datasets of isotopic ratios or chemical compositions^38–42^. In the geochemical context, the extracted IC vectors indicate specific directions along which the chemical compositions of the samples shift independently, regardless of the absolute elemental contents.

The ICA was applied to a 249 × 30 data matrix; the rows corresponding to the individual bulk sediment samples and the columns corresponding to the variables. The variables include the SiO_2_, TiO_2_, Al_2_O_3_, Fe_2_O_3_, MnO, MgO, CaO*, Na_2_O, P_2_O_5_, ΣREY, Sc, V, Cr, Co, Ni, Cu, Zn, Rb, Sr, Zr, Nb, Mo, Ba, Hf, Ta, Pb, Th, U, and CaCO_3_ concentrations and bulk carbon isotopic ratio (δ^13^C). The K_2_O content was not included in the matrix because a large number of the samples showed K_2_O values below the detection limit^32^; ΣREY represents the total content of rare-earth elements and yttrium (REY). In addition, to avoid a redundancy of information, we defined and used CaO*, the CaO content from non-carbonate fraction, that is calculated from total CaO and CaCO_3_ contents (see Methods). The multi-elemental data demonstrate a clear non-Gaussianity reflected in multiple elongated structures or minor clusters in the compositional space (Supplementary Fig. S2), which are far from the theoretical ellipsoidal data structure of a multivariate Gaussian probability distribution^29^.

By applying ICA to the 30-dimensional dataset, we extracted four ICs that collectively accounted for 83.9% of the total sample variance. Although it is an important parameter, the number of ICs to be extracted cannot be theoretically and uniquely determined. Alternatively, it is experimentally determined, considering the balance of ‘dimension reduction’ to exclude noise (e.g. analytical error) and ‘retention of original information’ in each case^29, 40, 42^. The extracted ICs cannot be ranked based on their proportion of data variance as in the case of common PCA, simply because the ICs are mutually independent and thus equivalently important. Therefore, the numbering of ICs is interchangeable and does not indicate that, for example, IC1 is more important than IC2.

When the number of extracted ICs increases from four to five, the proportion of the sample variance collectively accounted for by the new five ICs slightly increases to 87.2% (Supplementary Table S1). Subsequently, one of the original four ICs, the carbonate-dominated IC2 (see below), is further separated into two ICs. However, they show only slight differences and do not provide an essentially new interpretation of the data structure. Therefore, we consider that the original four ICs sufficiently extract the fundamental features of the dataset.

**Figure 3:**
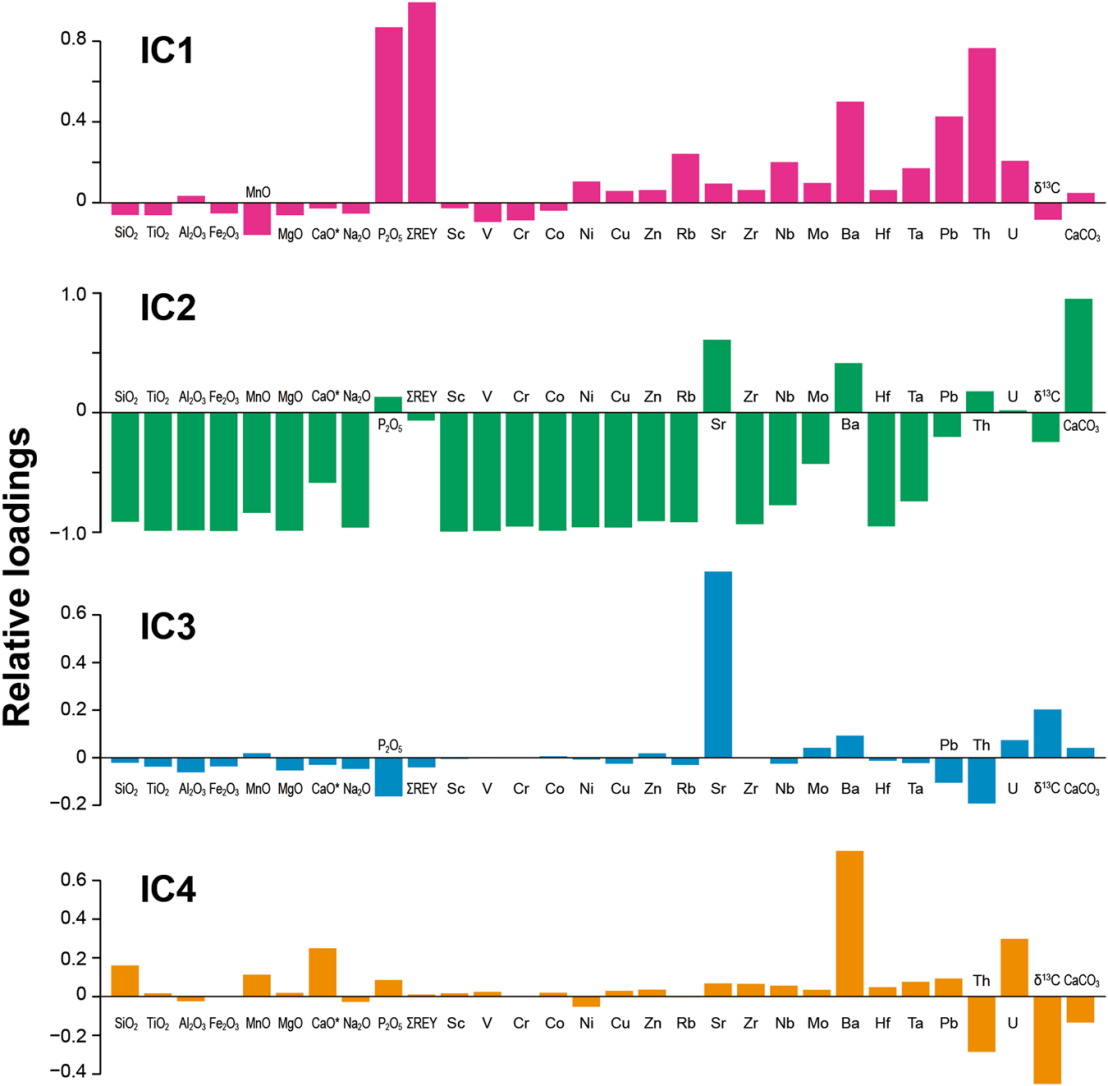
Relative loadings of each variable for IC1 to IC4. The relative loading of the *j*-th variable for the *i*-th IC can be obtained by dividing *a*_*ij*_ or the (*i*, *j*) component of the estimated mixing matrix A (see Methods) by a sample standard deviation of the *j*-th element.

**Figure 4:**
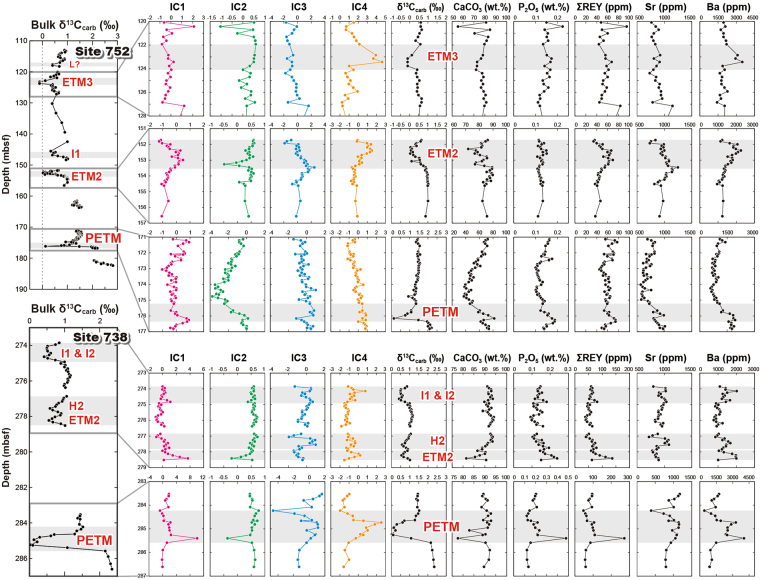
Depth profiles of the extracted ICs and the representative geochemical variables across multiple hyperthermals at the ODP Sites 752 and 738. The leftmost panels show the entire profiles of bulk carbonate δ^13^C values (δ^13^C_carb_) at each site and the other small panels provide the enlarged views of the events. The grey-shaded intervals indicate the negative CIEs corresponding to the PETM and Eocene hyperthermals.

We demonstrate the ICA result by using IC loadings and IC scores (Figs 3 and 4; Supplementary Figs. S2 and S3). Their definitions have already been given in detail elsewhere^42^. The IC loadings represent the relative contributions of each original variable to individual geochemical ICs, which correspond to the estimated mixing matrix. The IC scores are the coordinate values of the sample data in the IC space of the statistically independent base vectors or ICs.

To check the statistical robustness of the ICs, factors that potentially affect the ICA results, such as outliers in the dataset or the variability of the sample subsets between the studied sites, were further investigated (Supplementary Figs S4−S8). The results of the sensitivity tests indicated that the geochemical ICs extracted in this study were statistically robust and valid.

## Discussion

The statistically independent trends in the chemical compositions that are extracted as the geochemical ICs correspond to individual source materials and/or processes that originated the deep-sea sediments. By focusing the elements with large IC loading, we can effectively characterise each IC in the geochemical context and can interpret its geological and palaeoceanographic implications.

The IC1 shows distinct positive loadings of P_2_O_5_, ΣREY, and Th (Fig. 3). This indicates that the IC1 extracts the influence of biogenic calcium phosphate, relatively enriched in REY and Th, on the bulk composition of deep-sea sediments^43–45^. Although the absolute values of the Th content are very low in our bulk samples (generally < 1.5 ppm; ref. 32), the ICA extracts a strong positive correlation between these elements.

Biogenic calcium phosphate constitutes bones and teeth of marine vertebrates such as fish and is a common component of deep-sea sediments. In particular, it significantly accumulates in pelagic clay that deposits quite slowly^32, 42, 46^. Figure 4 shows the time-series (i.e. downhole) variations of the IC scores. Because the IC1 profiles do not mirror the profiles of the CaCO_3_ content, IC1 does not show apparent relationship that is simply caused by the decrease in the sedimentation rate or the reduction of the dilution effect of carbonate. It might rather reflect another environmental factor involving the net increase of biogenic calcium phosphate deposition in the studied region.

The small δ^13^C loading of the IC1 indicates that the abundance of biogenic calcium phosphate in the studied samples is essentially unrelated to the δ^13^C variation. In other words, it is unlikely that the phenomenon corresponding to IC1 is controlled by the episodic hyperthermals. Alternatively, the IC1 signal possibly reflects a net change in biomass of marine vertebrates^47^ or a migration of fish assemblages to the studied region.

The IC2 shows positive CaCO_3_, Sr, and Ba loadings and negative loadings of most of the other elements (Fig. 3). The elements Sr and Ba are alkaline earth metals and thus behave similar to Ca, resulting in the simultaneous enrichment of these elements in calcareous sediments. The negative CaO* loading indicates the variation in Ca contents of the bulk sediment associated with the non-carbonate detrital materials. The entire feature of the IC2 loadings appears to be a mixing relationship between CaCO_3_ and other components, including detrital materials, such as aeolian dusts and volcanic ashes, within these carbonate-dominated sediments. This relationship can be clearly seen in the diagrams presenting IC2 scores and elemental contents associated with detrital aluminosilicates (e.g. Al_2_O_3_, TiO_2_, and SiO_2_; Supplementary Fig. S9). However, the difference within the detrital materials does not explicitly show up in the present ICA results, because the compositional variations in the aluminosilicates, compared to the other components in the present dataset, are not large enough to be extracted clearly and robustly^42^. For a more detailed separation of these components (e.g. aeolian dust and volcanic ashes, or their compositional changes), a more comprehensive dataset containing larger compositional variations is required. To summarise, IC2 reflects the significant dilution effect of biogenic calcium carbonate on other elemental contents as one of the major structures in our dataset.

The IC3 shows a distinct positive Sr loading (Fig. 3), indicating that this IC largely reflects the Sr content variation. Generally, a first-order controlling factor for Sr in deep-sea sediment is its substitution for Ca in carbonates^46^. However, this feature appears to be already extracted as a positive Sr loading in the IC2. The Sr contents of our samples could be potentially affected by other factors including volcaniclastic detritus^46^, Sr-rich aragonite^46^, some types of radiolaria having a skeleton composed of SrSO_4_ (called Acantharia group), variation of the Sr/Ca ratio in biogenic carbonate due to changes in the species assemblage of calcareous nannoplankton^48, 49^, or authigenic calcite overgrowth having a lower Sr/Ca ratio than that of biogenic calcite constituting nannofossils^49, 50^.

The typical Sr contents of the Kerguelen Islands lavas and Broken Ridge basalt are 200–400 ppm^51^. These values are insufficient to explain bulk Sr contents of 390–1,200 ppm at Site 738 and 570–1,300 ppm at Site 752 without significantly affecting the bulk compositions of other elements (see also the Supplementary text and Fig. S10). Carbonate sediments containing abundant aragonite have high concentrations of Sr (2,000–4,000 ppm; ref. 46) and thus high Sr/Ca ratios (>3 mmol/mol), which is inconsistent with our samples (<1,300 ppm and 0.8–1.8 mmol/mol, respectively). Indeed, an X-ray diffraction (XRD) analysis on the representative samples showing high Sr content resulted in no detection of aragonite peaks (Supplementary Fig. S11). Acantharia is a relatively common radiolarian group that is widespread in the present surface ocean. However, their skeleton composed of SrSO_4_, is easily dissolved during its postmortem deposition on the seafloor and thus, is not preserved in the sediment. Therefore, it is unlikely that the labile skeleton significantly contributes to the Sr content in the bulk sediment composition.

The bulk Sr/Ca ratio in calcareous nannofossils can vary depending on the fractions of species that favour oligotrophic, mesotrophic, or eutrophic conditions in the surface ocean. During the Eocene hyperthermals, the temporal Sr/Ca ratio increases in the bulk coccolith from the Walvis Ridge were influenced by variations in the species assemblage compositions reflecting a productivity optimum^49^. It was suggested that minor fluctuations of the bulk δ^13^C values during the PETM could be explained by taxonomic variations in the nannoplankton assemblages with different δ^13^C values^52^. Therefore, if IC3 involves the change of nannofossil assemblages, a modest positive δ^13^C loading of IC3 might indicate a vital effect on the bulk carbon isotopic signature across the shift of dominant taxa.

In addition, variations in the degree of secondary calcite overgrowth also significantly affect the bulk Sr/Ca ratio of calcareous nannofossils. Biogenic marine calcites have much higher Sr partitioning coefficients than abiogenic secondary calcites and thus, each bulk carbonate sample is a mixture of abiogenic, secondary calcites with low Sr/Ca ratios (~0.13 mmol/mol; ref. 53) and pristine nannofossils with relatively high Sr/Ca ratios (1.5–2.0 mmol/mol; ref. 50). Therefore, the bulk Sr/Ca ratio increases when the secondary calcite overgrowth on primary nannofossils is small, which can be caused by the transient bottom water that was much less saturated (or even undersaturated) with respect to CaCO_3_ during the hyperthermals^49^.

In general, the IC3 scores positively shift during the CIE intervals (Fig. 4). Because the two possible processes, the change of nannofossil assemblage composition and the decrease of abiogenic calcite overgrowth due to ocean acidification, are not mutually exclusive, both can cause the Sr to increase in the bulk chemical compositions of the studied sediments during the hyperthermals. On the other hand, it was reported that such a variable diagenetic process involving overgrowth did not substantially alter δ^13^C signatures of the Palaeogene carbonates across the hyperthermal events^50^. Hence, we consider that our δ^13^C data certainly maintain the original signatures recorded in the pelagic carbonate sediment during the late Palaeocene–early Eocene period.

The IC4 is characterised by both positive Ba and negative δ^13^C loadings (Fig. 3). When δ^13^C decreases (i.e. negative CIEs during hyperthermals), the Ba content in the sediment tends to increase. This feature strongly suggests that IC4 corresponds to an increase of the marine barite (BaSO_4_) accumulation due to an increased export productivity^54^, which efficiently sequestered the surplus carbon in the ocean–atmosphere system and terminated the hyperthermal events^11, 15, 55^. The barite accumulation rate (BAR) is often calculated to evaluate the changes in the past export productions^11, 15^. In the present study, however, we discuss the signature of the change using the IC that was statistically derived from the compositional data because the ICA can extract the compositional shift regarding the barite deposition that is independent of the dilution effect of carbonate.

The IC4 scores shift positively during the CIEs, especially in their recovery phases (Fig. 4). This means that the biogeochemical feedback commonly played an important role in the recovery from short-term climatic aberrations in the early Palaeogene greenhouse environment. During the recovery phase of the PETM, continental chemical weathering intensified due to the warm and humid climatic conditions^10^. The accelerated hydrologic cycle significantly increased the terrestrial runoff and consequently, the nutrient delivery to the ocean. This should have stimulated the surface ocean productivity, which resulted in atmospheric CO_2_ drawdown and quiet down of extreme warming^2, 11, 13, 15^. While this productivity feedback has been reported from the PETM horizon in a number of previous studies^11, 13, 15^, we, in our study, demonstrate that the Earth system’s negative feedback also functioned in a similar manner during multiple other smaller hyperthermals in the Indian Ocean, including the ETM2/H2, I1/I2, and ETM3 events (Fig. 4). However, no clear IC4 peak could be recognised in the PETM interval at Site 752. This might be due to the influence of significant volcanic ash contamination^30^ that could have obscured the original signature, especially with respect to Ba. Alternatively, as a local feature, the export production indeed did not increase during the PETM at this site^56^.

**Figure 5:**
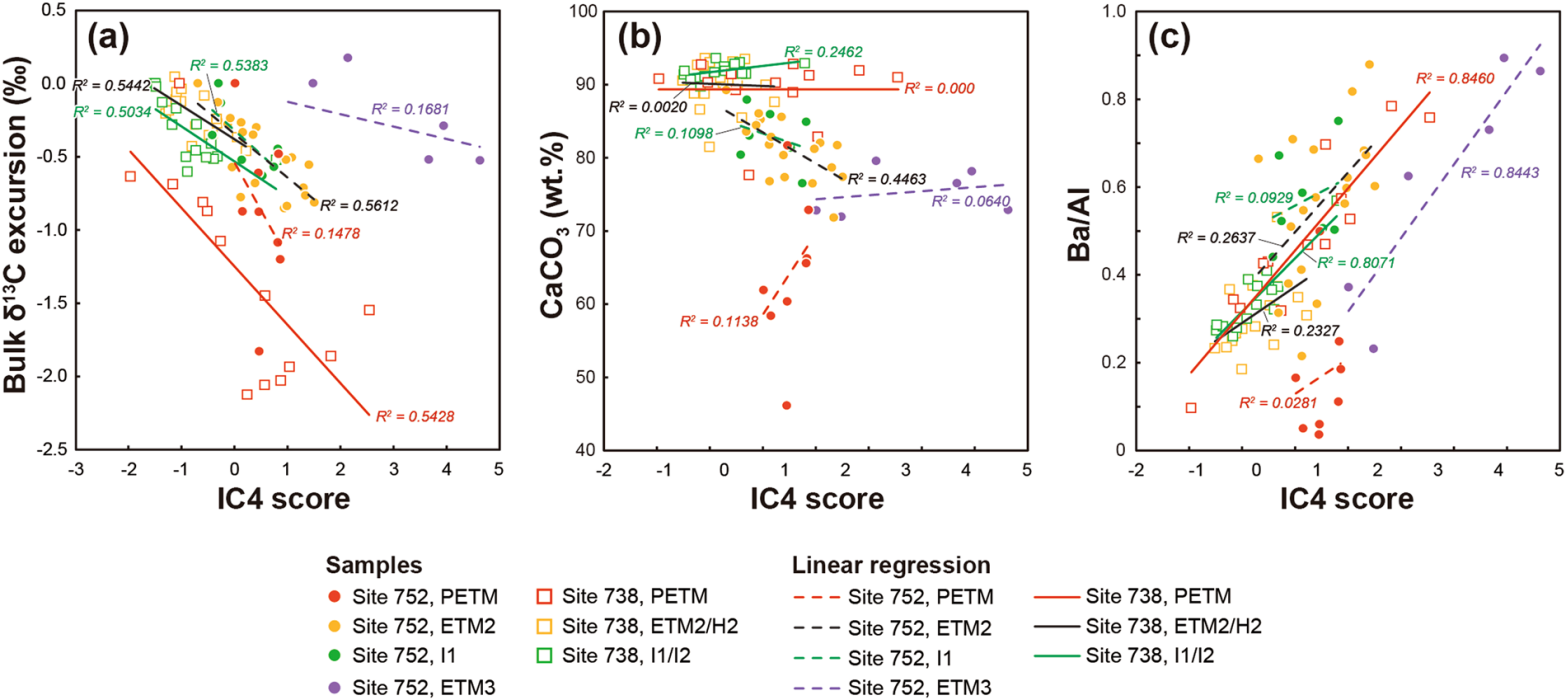
Relationships between IC4 and the geochemical variables. IC4 score versus (**a**) bulk δ^13^C excursion, (**b**) CaCO_3_ content, and (**c**) Ba/Al ratio. The data plotted are from the intervals of the hyperthermal events shown in Figs 2 and 4. The linear regression lines and their determination coefficients (R^2^-values) for the individual hyperthermal intervals are also presented.

To further clarify the IC4 features, the relationships between the IC4 score and the bulk δ^13^C excursion, CaCO_3_ content, and Ba/Al ratio are shown for the individual hyperthermal intervals (Fig. 5). The length of the linear regression line for each hyperthermal interval reflects the variations in the IC4 and three geochemical variables, which basically corresponds to the magnitude of each event, except for the PETM at Site 752. The IC4 is inversely correlated with the bulk δ^13^C excursion (Fig. 5a), whereas the IC4 and CaCO_3_ contents are generally uncorrelated (Fig. 5b). This indicates that the IC4 certainly characterises the response of the carbon cycle during the hyperthermals but is not affected by the carbonate dissolution. Moreover, to eliminate the influence of the changes of the carbonate content or sedimentation rate, the Al-normalised Ba content is plotted against the IC4 score (Fig. 5c). With increasing IC4 score, the Ba/Al ratio generally increases during the hyperthermals. Given that the Al content represents the contribution of terrigenous material supplied as aeolian dust to the open ocean sites, the increase of the Ba/Al ratio exhibits the increase of non-detrital Ba, that is, biogenic barite. Thus, although the Ba/Al ratio is still not an absolute but a relative index of the export productivity changes, the positive correlations between the IC4 and Ba/Al ratios of each hyperthermal interval demonstrate that the IC4 certainly extracts the net increase of biogenic barite accumulation as a result of enhanced export productivity.

The ETM3 at Site 752 deviates from the overall trend between the IC4 and CIE (Fig. 5a). Because the amplitude of the ETM3-CIE (−0.5‰) is comparable to that in other locations (Fig. 1b), the deviation originates from a relatively great deposition of excess Ba with respect to the amplitude of the CIE (Figs 4 and 5c). The paucity of data variations prevents us from further discussing if this feature is local (only in the site) or temporal (only during the ETM3), or both. Nevertheless, because the ETM3 and other hyperthermals have a similar trend in the IC4–Ba/Al diagram, we can generally conclude that an essentially identical feedback mechanism might have functioned during each Eocene hyperthermal event and in the PETM, although the magnitudes of the carbon cycle perturbations are even smaller than those of the PETM.

Previous studies have reported that significant population changes of calcareous nannoplankton in the tropical Pacific^57^ and Atlantic Oceans^58^ occurred when the environmental perturbation surpassed a certain level, for example, CIEs greater than 0.6‰, equivalent to ~2 °C of global warming^57^. This indicates that marine plankton ecosystem disruptions show a threshold behaviour closely depending on the amount of carbon released across each hyperthermal event. However, the direct relationship between the faunal change and productivity feedback via an enhanced biological pump has been poorly constrained. The behaviour of IC4 indicates that a productivity feedback mechanism operated when the Earth’s surface carbon cycle was perturbed (Figs 4 and 5c), at least in the southern Indian Ocean. This implies that the threshold-based faunal change in response to an environmental upheaval is, at least partially, decoupled from the Earth’s intrinsic negative feedback mechanism that sequesters the surplus carbon and recovers the system to an original steady state, regardless of the magnitude of the perturbation.

The profiles of IC3 and IC4 look nearly identical to those of the original Sr and Ba contents, respectively (Fig. 4). Given that these elements also dominate the relative loadings of these ICs (Fig. 3), one may consider that the information indicated by these ICs could likely be replaced by that provided by these elements and that there is no difference between the conclusion based on the ICs and the original elemental data. However, a conclusion based on the characteristic data structure extracted by a multivariate statistical analysis is essentially different from one focusing on the elements that are selected empirically or arbitrarily. In other words, the ICs with a small number of prominent loadings merely demonstrates that the important features of the sediments can be approximated well by these limited elements (e.g. Sr or Ba) as an outcome of a thorough consideration with no *a priori* intuition. Hence, the ICs can be a novel and integrative barometer that reflects the entire information, or the whole data structure, of all the variables (i.e. the elements and isotopes) analysed.

Our new data and analyses of the Indian Ocean sediment shed new light on the biogeochemical processes across transient global warming episodes in the early Palaeogene. Further integration of isotopic signatures and multi-elemental variations from other regions and times on Earth and a comprehensive analysis from palaeontological, sedimentological, and statistical perspectives can verify if the geochemical ICs characterised in the present study are merely local/temporal features or globally/secularly robust characteristics of the Palaeogene greenhouse world or Cenozoic Earth.

## Methods

### Constraints on the age of samples

We sampled and analysed 173 samples from Holes 752 A and 752B between 113.0 and 182.4 m below the seafloor (mbsf). The biostratigraphic records indicate that this interval corresponds to the latest Palaeocene–earliest Eocene (Fig. 2). According to a previous report of calcareous nannofossil records^59^, the first occurrence of *Discoaster multiradiatus* was between 203.58 and 202.08 mbsf, the last occurrence of *Fasciculithus* spp. was between 174.58 and 171.58 mbsf, and the last occurrence of *Tribrachatus contortus* was between 145.48 and 142.48 mbsf, corresponding to the nannofossil zones CP8a/CP7 boundary at 57.21 Ma, CP8b at 55.64 Ma, and CP9b/CP9a boundary at 54.17 Ma, respectively^37^. Regarding the magnetic polarity, C24r/C25n boundary at 57.10 Ma^37^ was identified within the interval of 201.64–200.51 mbsf^59^ (Fig. 2). The assignment of the long reversed polarity between 201 and 155 mbsf to C24r was supported by the first occurrence of *Tribrachiatus bramlettei* at 171.1 mbsf^30^. In the shipboard report^30^, the overlying normal polarity from 155 to 135 mbsf was collectively described as ‘C24N-2’, and the following (upward in the core) reversed and normal polarities from 135 to 127 mbsf and 127 to 120 mbsf were described as ‘C24R-1’ and ‘C24N-1’, respectively. In the present compilation of biomagnetostratigraphy^37^, much shorter magnetic reversals were inserted in the previous ‘C24N-1’ and thus, the chron C24 was reorganised into C24n.1n, C24n.1r, C24n.2n, C24n.2r, C24n.3n, and C24r in a descending order^37^. Considering that the nannofossil zone CP9 overlaps C24n.3n^37^ and that the duration of C24n.2r was only 0.08 Myr^37^, we assigned the positive polarities from 155 to 135 mbsf as C24n.3n, although the assignment of all normal polarities in the interval contains uncertainties due to potentially missing information due to the core gaps (Fig. 2). Although arbitrary, considering the presence of a relatively large CIE during the normal polarity between 127 and 120 mbsf, we assigned this normal polarity as C24n.1n that contains the ETM3 event (Fig. 1b), and the underlying reversed polarity as C24n.1r. The nannofossil zone CP10/CP9b boundary at 115.05 mbsf^30, 59^, based on the first occurrence of *Discoaster lodoensis*, was somewhat inconsistent with the magnetic polarity records; C23r/C24n.1n boundary was at 52.62 Ma, whereas CP10/CP9b boundary at 53.70 Ma^37^. Between the two, we prefer the magnetostratigraphic constraint because of the consistency with the negative CIEs.

We sampled and analysed 76 samples from Hole 738 C between 235.2 and 286.6 mbsf (Fig. 2). Based on the calcareous nannofossil records^60^, the first occurrence of *Discoaster multiradiatus* was between 312.30 and 293.00 mbsf, the first occurrence of *Discoaster diastypus* was between 284.30 and 283.40 mbsf, the first occurrence of *Discoaster lodoensis* was between 264.76 and 264.10 mbsf, and the first occurrence of *Discoaster sublodoensis* was between 227.79 and 226.26 mbsf, corresponding to the boundaries of CP8a/CP7 at 57.21 Ma, CP9a/CP8b at 54.95 Ma, CP10/CP9b at 53.7 Ma, and CP12a/CP11 at 49.1 Ma, respectively^37^. At this site, although there was no palaeomagnetic control because of large core gaps^31^, the planktonic foraminifera zones for high latitudes were reported^35^; AP5/AP4 boundary (Palaeocene-Eocene boundary) at ~285 mbsf and AP5b/AP5a boundary at ~278 mbsf. The AP5 zonation corresponds to the low-latitude foraminifera zone P6b between 54.61 and 52.54 Ma^37^.

### Geochemical and mineralogical analyses

Sediment samples were collected using 5 or 10 cm^3^ scoops. The bulk sediment samples were dried at 40 °C and powdered in an agate mortar prior to the chemical analyses. Major and trace element contents were determined using an X-ray fluorescence (XRF) spectrometer and an inductively coupled plasma mass spectrometer, respectively, both at the Department of Systems Innovation, School of Engineering, at the University of Tokyo. The sample preparation methods, analytical procedures, and analytical results of the major and trace element compositions were already reported by Yasukawa *et al*.^32^ In this study, we additionally obtained major and trace element data for 75 samples across the PETM intervals (Supplementary Data S3 and S4) and combined them with the published data^32^.

The bulk carbon isotopes of the powdered samples were measured using a GV Instruments IsoPrime with a Multicarb preparation system (Wythenshave, Manchester, United Kingdom) at the Center for Advanced Marine Core Research, Kochi University. The measured isotopic ratios were converted to standard delta notation relative to Vienna Pee Dee Belemnite (VPDB). Repeated measurements (*n* = 46) of the NBS-19 standard demonstrated that the analytical precision (2σ) was better than ±0.06‰.

The bulk CaCO_3_ content was determined with coulometric titration using a CO_2_ coulometer (UIC Inc., Coulometrics model CM5012) at the Center for Advanced Marine Core Research, Kochi University. Approximately 5 mg of the powdered sample reacted with 10% H_3_PO_4_ for 15 min and the released CO_2_ gases were analysed with the coulometer. The analytical precision (2σ) of the measurement was ±2.3 wt.% based on the repeated measurements (*n* = 71) of an in-house CaCO_3_ standard.

We define a new variable CaO* as the CaO content from the non-carbonate fraction calculated by subtracting the CaO content of the carbonate fraction, that is converted from the measured CaCO_3_ content, from the total CaO content obtained by the XRF analysis. Regarding the carbonate fraction, we use the original data of the bulk CaCO_3_.

XRD analysis was performed using a Rigaku Ultima IV X-ray diffractometer at the Department of Systems Innovation, the University of Tokyo, with Cu Kα1 radiation at an accelerating voltage of 40 kV and a tube current of 40 mA. Samples were powdered in an agate mortar and placed on a slide. XRD measurements were carried out at a scanning speed of 2θ = 4°/min (step width: 2θ = 0.02°) over a range of 2θ = 3°−70°.

### Independent Component Analysis

Here, we assume that each sample datum can be expressed as a linear mixture of several geochemical ICs that are mutually independent. We also assume that each IC shows non-Gaussian features (i.e. the probability density or the data histogram along the IC deviate from the Gaussian distribution), which means that each IC is statistically informative and not a random signal such as white noise. Based on these assumptions, we can numerically solve the linear ICA model as follows:

1 $${\bf{X}}={\bf{S}}{\bf{A}},$$

where **X** is the original data matrix composed of 249 samples × 30 variables (elemental contents and δ^13^C), **S** is an independent source matrix whose columns correspond to the ICs, and **A** is the linear mixing matrix. The mathematical principles and numerical methods of the ICA have been reported elsewhere^29, 40, 42^. In the present work, we first transformed the data **X** to have zero mean and unit variance along the principal components using a basic PCA algorithm. After this ‘whitening’ process, any pair of new variables is mutually uncorrelated but not necessarily independent. Subsequently, the whitened axes were rotated until the projection of the whitened data on each axis provided histograms with maximum non-Gaussianity. We evaluated the non-Gaussianity by negentropy *J*(*y*) approximated as follows:

2 $$J(y)={[{\rm{E}}\{G(y)\}-{\rm{E}}\{G(\nu )\}]}^{2}$$

3 $$G(y)=\,\mathrm{log}\,\cosh (y),$$

where *y* is a random variable with zero mean and unit variance and *ν* is the Gaussian variable of zero mean and unit variance. The function *G* is selected, following Hyvärinen *et al*.^29^, to appropriately capture marginal structures or minor features of the data, such as peaks deviating from a general trend, originating from short-term and prominent environmental perturbations. We employed the FastICA algorithm^29^ to implement the above-mentioned numerical processes and utilised a modified open R package^61^ to perform the calculation.

### Data availability

The data that support the findings of this study are available from the corresponding author upon request.

## Electronic supplementary material

Supplementary Information

Dataset 1
