# Supplementary material for: Earth system feedback statistically extracted from the Indian Ocean deep-sea sediments recording Eocene hyperthermals
Source: Sci Rep. 2017 Sep 12;7:11304. doi: 10.1038/s41598-017-11470-z (PMC5595800; doi:10.1038/s41598-017-11470-z)
Supplement: Supplementary file 1 — Supplementary Information [file 41598_2017_11470_MOESM1_ESM.pdf]

Earth system feedback statistically extracted from the Indian Ocean deep-sea sediments recording Eocene hyperthermals

Kazutaka Yasukawa[1,2,*], Kentaro Nakamura[1], Koichiro Fujinaga[2,3], Minoru Ikehara[4], Yasuhiro Kato[3,1,2,5,*]

[1] Department of Systems Innovation, School of Engineering, University of Tokyo, 7-3-1 Hongo, Bunkyo-ku, Tokyo 113-8656, Japan

[2] Ocean Resources Research Center for Next Generation, Chiba Institute of Technology, 2-17-1 Tsudanuma, Narashino, Chiba 275-0016, Japan

[3] Frontier Research Center for Energy and Resources, School of Engineering, University of Tokyo, 7-3-1 Hongo, Bunkyo-ku, Tokyo 113-8656, Japan

[4] Center for Advanced Marine Core Research, Kochi University, B200 Monobe, Nankoku, Kochi 783-8502 Japan

[5] Research and Development Center for Submarine Resources, Japan Agency for Marine-Earth Science and Technology (JAMSTEC), 2-15 Natsushima-cho, Yokosuka, Kanagawa 237-0061, Japan

*Corresponding authors:
Kazutaka Yasukawa
Department of Systems Innovation, School of Engineering, The University of Tokyo, 7-3-1 Hongo, Bunkyo-ku, Tokyo 113-8656, Japan
Telephone: +81-3-5841-7018
E-mail: k-yasukawa@sys.t.u-tokyo.ac.jp

Yasuhiro Kato
Frontier Research Center for Energy and Resources, School of Engineering, The University of Tokyo, 7-3-1 Hongo, Bunkyo-ku, Tokyo 113-8656, Japan
Telephone: +81-3-5841-7022
E-mail: ykato@sys.t.u-tokyo.ac.jp

Possibility for diagenetic alteration

In general, our bulk $\delta^{13}C$ values are within the range of pelagic bulk carbonate records reported by previous studies using comparable Deep Sea Drilling Project/ODP cores (typically between +4 and 0‰; Refs. S1–S4) throughout the studied intervals, and thus, we do not consider that our samples were affected by severe diagenetic alterations. As a possible process that affects the original geochemical signatures, it was suggested that the $\delta^{13}C$ record in the sediments could be significantly altered by the post-depositional carbonate precipitation with a breakdown of organic matter (Ref. S1). If it actually occurs, the bulk carbonate $\delta^{13}C$ shows a very low value (~ −20‰). Such abnormal data were not identified in our dataset.

The post-depositional overgrowth in the carbonate ooze is controlled by the carbonate saturation state in the bottom water and/or the pore water, and the degree of the diagenetic overgrowth affects the Sr/Ca ratio of the nannofossils (Ref. S5). However, it was reported that such a variable diagenetic process involving overgrowth did not substantially alter $\delta^{13}C$ signatures of the Palaeogene carbonates across the hyperthermal events (Ref. S5). Hence, we consider that our $\delta^{13}C$ data certainly maintain the original signatures recorded in the pelagic carbonate sediments during the late Palaeocene–early Eocene period, and also that the diagenetic alteration of Sr/Ca could be extracted as an independent compositional change (i.e. IC3 in the main text) by the ICA.

Checking the statistical robustness of the independent components

Effect of outliers in the dataset

In this study, to capture marginal structures or minor features of the data that originated from short-term and prominent environmental perturbations (e.g. peaks deviating from a general trend), we chose the *logcosh* function as the evaluation function of non-Gaussianity (i.e. $G(y)$ in 'Fundamentals of ICA' in the Methods section) because it reflects the characteristic structure both around the centre (mean) and the marginal regions ('tails' in data distributions) of the data, and thus, is regarded as a good general-purpose function (Ref. S6).

We checked the robustness of our ICA results by using a limited dataset, excluding the samples with high values for each IC (greater than 3) in the original results. Because each IC is constrained to have unit variance in the fastICA algorithm (Ref. S6), an IC score of one corresponds to unit standard deviation (1σ) along each IC. Thus, samples

having absolute values of IC scores greater than three constitute the long tails outside the 3σ range in the IC score distributions that have strong non-Gaussianity such as a large skewness and kurtosis (Supplementary Fig. S3). We define an 'outlier' as a sample that has an IC score greater than three in absolute value for any IC (Ref. S7).

The ICA result with no outliers (n = 236) had essentially identical features to the original result (Supplementary Figs. S4 and S5). Although the IC4 loadings of the elements other than Ba and $\delta^{13}C$ became larger than the original result, the depth profiles of IC4 showed almost the same patterns. Therefore, this test confirms that the geochemical ICs derived from all data certainly reflect the fundamental structure in the dataset and are not artefacts generated by a small number of outliers.

Effect of the difference in sample subsets between the Sites 752 and 738

We further evaluated how ICA results change when each site is treated as separate populations to analyse. In the ICA result using only the Site 752 samples (n = 173), the depth profiles of IC scores partly differed from the original result (Fig. 4 and Supplementary Fig. S8a) because the loading values changed moderately (Fig. 3 and Supplementary Fig. S6). However, the fundamental features or characteristic elements for each IC were essentially unaltered and sufficiently comparable to the original ones.

On the contrary, the ICA result using only the Site 738 samples (n = 76) showed somewhat complicated features (Supplementary Fig. S7). Although the ICs corresponding to the original IC3 and IC4 were extracted relatively clearly, the IC1 with prominent loadings of P_2O_5 and ΣREY did not appear. Instead, the other two ICs were characterised by relatively large $CaCO_3$ loadings, inverse to most of the other variables. We assigned the IC with large P_2O_5 and ΣREY loadings as IC1 on the basis of the significant peak near the base of the PETM in the depth profiles (Fig. 4 and Supplementary Fig. S8b). The remnant IC was assigned as IC2 and its depth profile showed a comparable pattern to the original IC2. Such a somewhat ambiguous IC1resulted, at least partly, from a much smaller sample size at the Site 738 which was not large enough for an effective statistical analysis on the high-dimensional dataset.

Supplementary Fig. S1 shows histograms of elemental contents and IC scores for the Sites 752 and 738 (the IC scores are estimated from the entire dataset). The distributions of the original data in each site generally overlapped, indicating a common data structure. However, several elements show different distribution patterns. For

example, MnO and $CaCO_3$ show distinct peak positions between the two sites. Distributions of Th contents did not overlap. Moreover, the long tails in the distributions of most elements are dominated by Site 752 samples. These differences of the original data structure affect the ICA results when the data from each site are treated separately.

Although partly different features were recognised, the main structures of the data appeared to be common to data subsets from the two sites. Thus, we discussed the general processes regarding the hyperthermals on the basis of the entire sample set in the main text.

Relationships between IC3 and Sr, and IC4 and Ba

IC3 and IC4 have prominent loadings of Sr and Ba, and thus, the depth profiles of IC3 and IC4 are very similar or almost identical to those of Sr and Ba, respectively. To characterise the similarities between these ICs and the elemental profiles, these elemental contents were plotted against the relevant IC scores (Supplementary Fig. S10).

In Fig. S10 (a), a sharp edge in the upper end of the data distribution reflects a strong linearity or similarity in the two variables. Figure S10 (a) also demonstrates that the samples from the Site 752A–Core 19 (including the PETM interval and also contaminated by volcanic ash) deviate towards lower Sr content from the overall linear trend. Moreover, the IC3 scores of the deviated samples are scattered around zero (typically −1 to +1) which supports hypothesis that the origin of IC3 is not a volcanic component because if a volcanic component originates the IC, the samples affected by volcanic ash necessarily have large absolute values of IC3 score.

In Fig. S10 (b), as well as Fig. S10 (a), all the data constitute a generally good positive correlation, although the samples from Site 752A–Core 19 slightly deviate towards lower Ba content from the overall trend. In addition, there is an obvious outlier showing a very high Ba content (> 4,000 ppm) in the PETM interval at Site 738 (Core 11–Section 2–49 to 51 cm, or 285.40 mbsf; see also Fig. 4 in the main text). The generally linear but relatively broad data distribution indicates that the correlation between IC4 and Ba can be recognised throughout the studied cores but is somewhat weaker than that between IC3 and Sr except for Site 752A–Core 19.

These features indicate that, except for the outlier, IC3 and IC4 profiles are

essentially similar to Sr and Ba contents, respectively, within all the intervals except for the Site 752A–Core 19 that is contaminated by volcanic ash.

XRD analysis to check for the contribution of aragonite

An XRD analysis was conducted on the representative samples showing high Sr content to check for the contribution of aragonite to the sediment samples (especially high Sr samples). The results indicate predominant calcite peaks in all the samples, whereas no aragonite peaks were detected (Supplementary Fig. S11). Hence, the possibility that Sr-rich aragonite attributed to the large loading of Sr for IC3 or the large variation of Sr content in the bulk chemical composition can be excluded.

Supplementary References

S1. Cramer, B. S., Wright, J. D., Kent. D. V. & Aubry, M.-P. Orbital climate forcing of $\delta^{13}C$ excursions in the late Paleocene–early Eocene (chrons C24n–C25n). *Paleoceanography* **18,** 1097, doi:10.1029/2003PA000909 (2003).

S2. Zachos, J. C., McCarren, H., Murphy, B., Röhl, U. & Westerhold, T. Tempo and scale of late Paleocene and early Eocene carbon isotope cycles: Implications for the origin of hyperthermals. *Earth Planet. Sci. Lett.* **299,** 242–249 (2010).

S3. Leon-Rodriguez, L. & Dickens, G. R. Constraints on ocean acidification associated with rapid and massive carbon injections: The early Paleogene record at ocean drilling program site 1215, equatorial Pacific Ocean. *Palaeogeogr. Palaeoclimatol. Palaeoecol.* **298,** 409–420 (2010).

S4. Slotnick, B. S. *et al.* Early Paleogene variations in the calcite compensation depth: new constraints using old borehole sediments from across Ninetyeast Ridge, central Indian Ocean. *Clim. Past* **11,** 473–493 (2015).

S5. Dedert, M. *et al*. Temporally variable diagenetic overgrowth on deep-sea nannofossil carbonates across Palaeogene hyperthermals and implications for isotopic analyses. *Mar. Micropaleontol.* **107,** 18–31 (2014).

S6. Hyvärinen, A., Karhunen, J. & Oja, E. *Independent Component Analysis*. (John Wiley & Sons, New York, 2001).

S7. Yasukawa, K., Nakamura, K., Fujinaga, K., Iwamori, H. & Kato, Y. Tracking the spatiotemporal variations of statistically independent components involving enrichment of rare-earth elements in deep-sea sediments. *Sci. Rep*. **6,** 29603, doi:10.1038/srep29603 (2016).

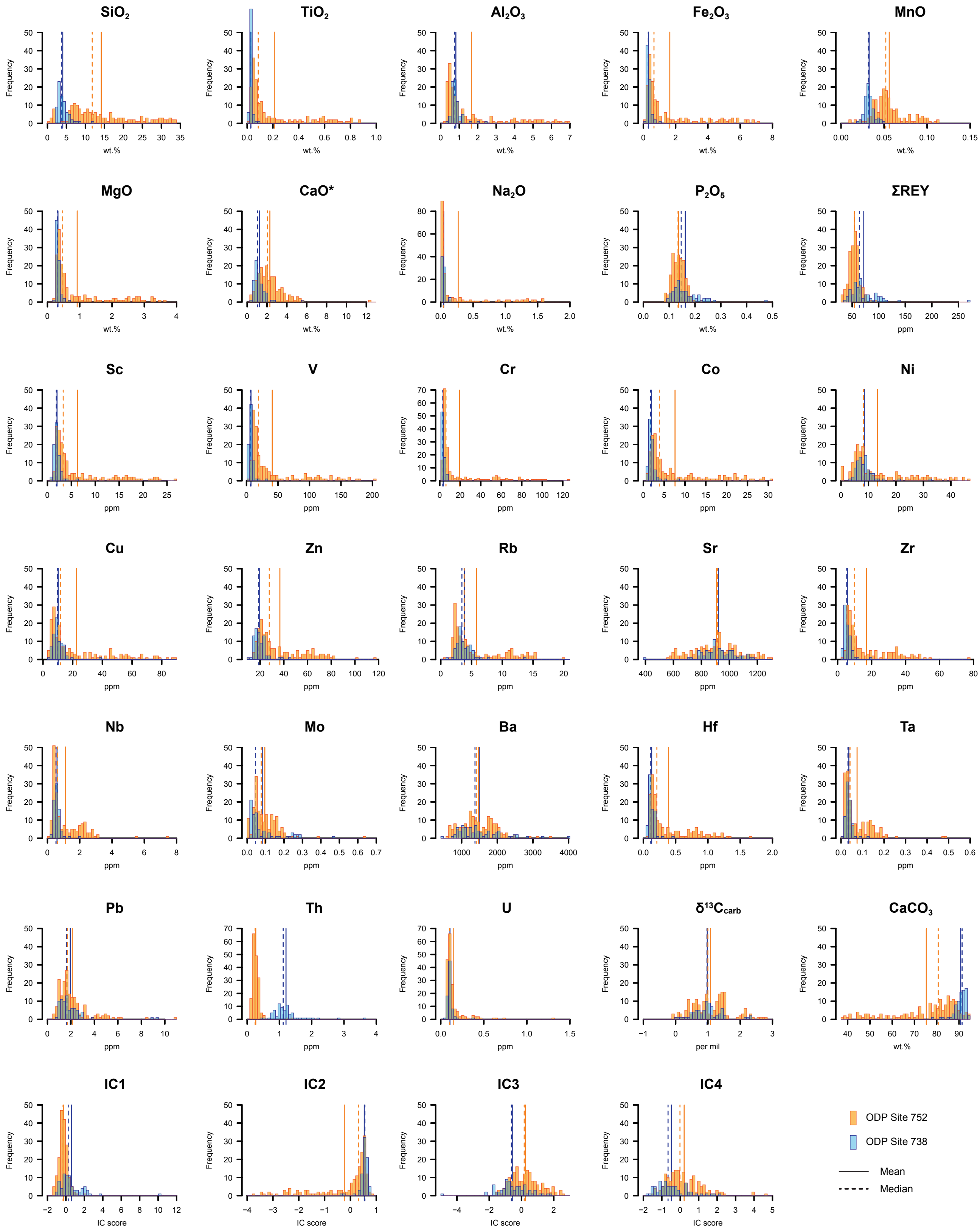

Supplementary Figure S1. Histograms of elemental content and IC scores derived from the bulk sediment samples collected from Sites 752 (orange) and 738 (blue). ICs correspond to the ICA results discussed in the main text. Vertical solid and dashed lines represent the mean and the median of each data distribution, respectively.

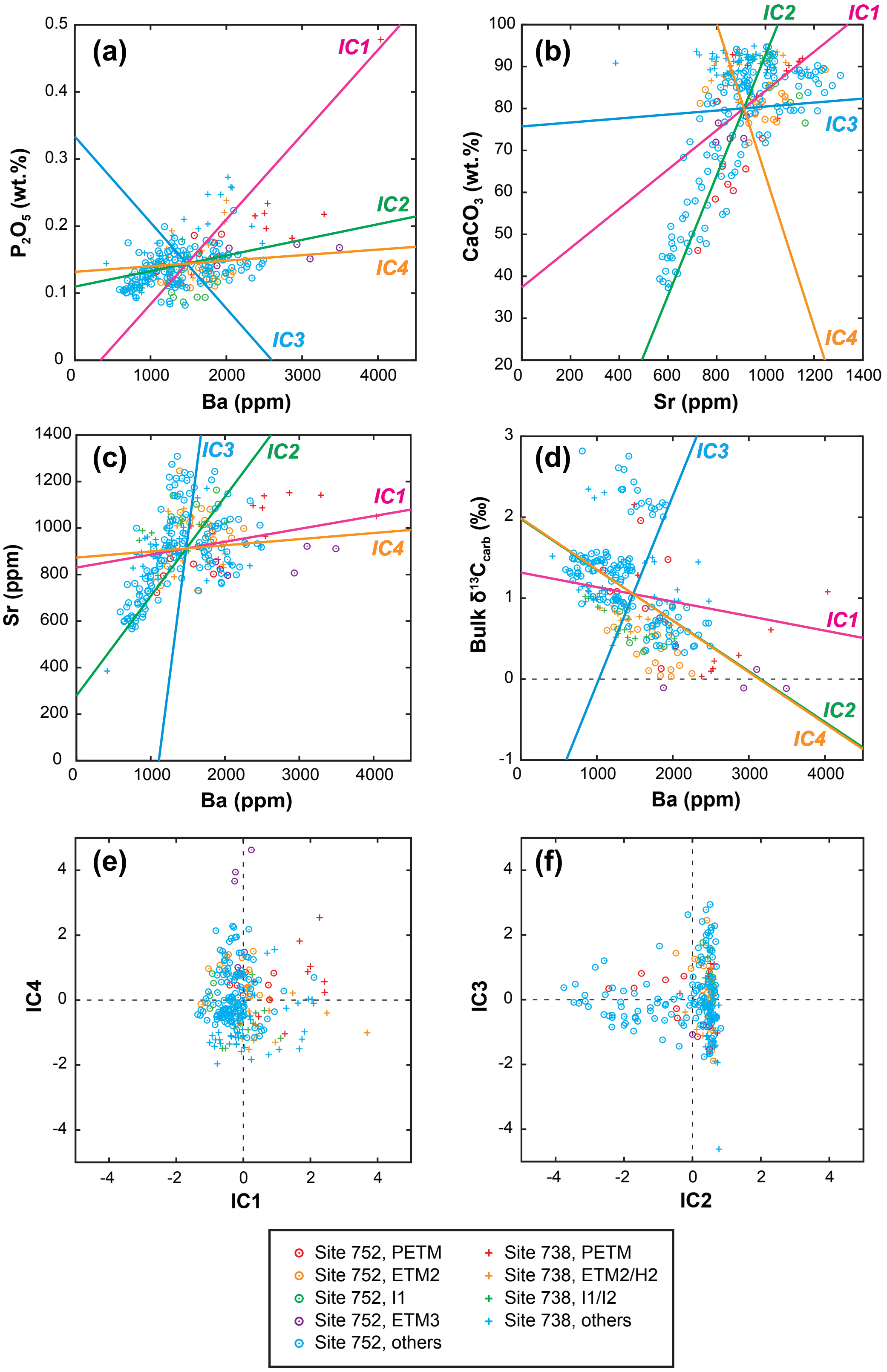

Supplementary Figure S2. Compositional data of the studied samples and extracted geochemical ICs. The ICs are projected in the representative 2-D compositional subspaces of (a) Ba–P_2O_5, (b) Sr–$CaCO_3$, (c) Ba–Sr, and (d) Ba–bulk $\delta^{13}C_{carb}$, as vectors showing specific directions along which the original chemical compositions or isotopic ratios shift. The label for each IC in each panel is placed along its positive axis. The original compositional or isotopic variables can be decomposed into the ICs and the data can be projected into IC subspaces such as the (e) IC1–IC4 and (f) IC2–IC3 spaces. The sample data from Sites 752 and 738 in each panel are shown as circles and crosses, respectively. The data during the hyperthermals (Figs. 2 and 4 in the main text) are colour-coded, as shown in the legend.

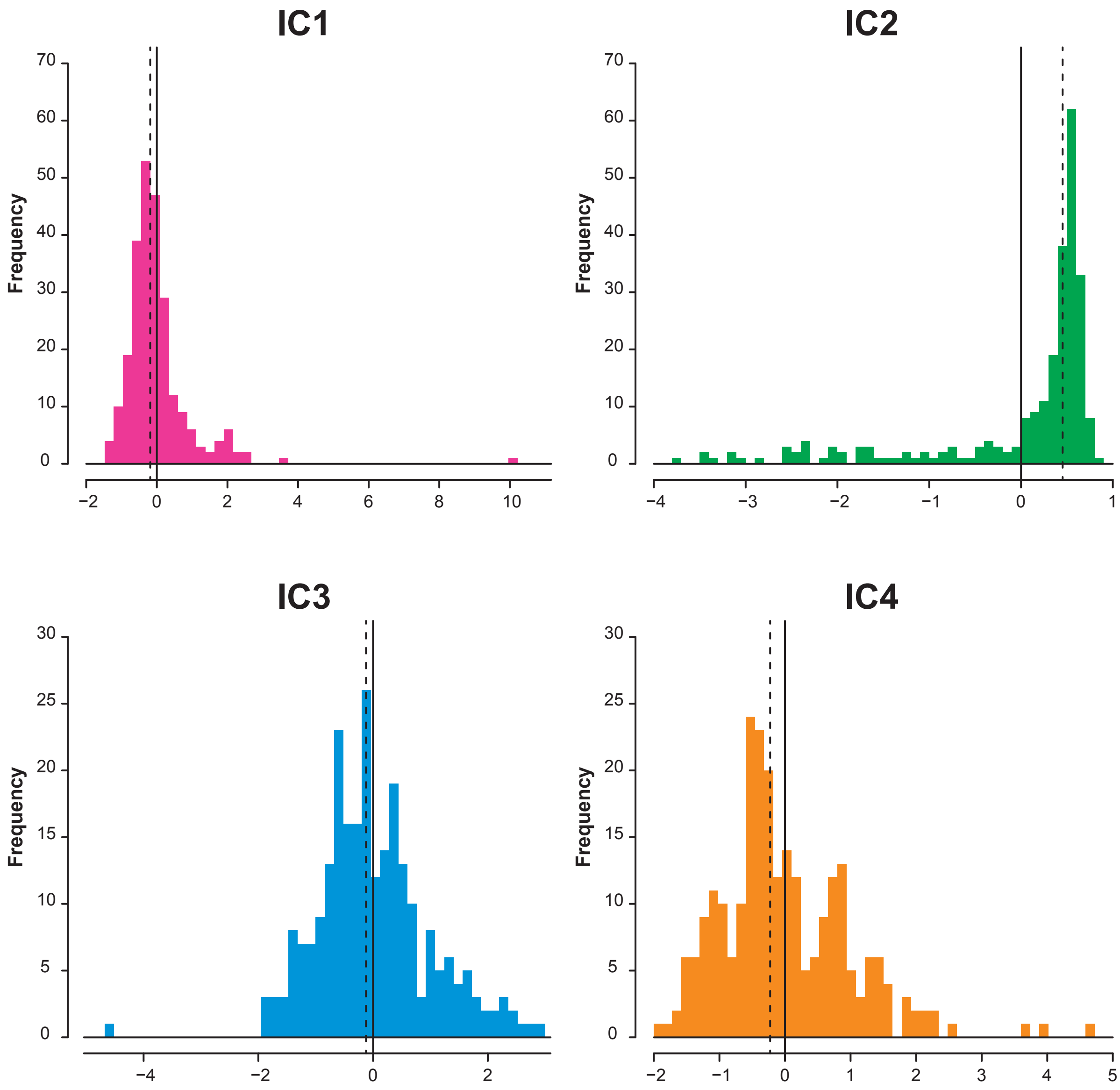

Supplementary Figure S3. Frequency distributions of the four ICs extracted in this study. The horizontal axis corresponds to each IC score. The vertical solid and dashed lines represent the mean and the median values for each IC, respectively. Each IC is constrained to have unit variance in the FastICA algorithm, therefore, an IC score of one corresponds to unit standard deviation along each IC.

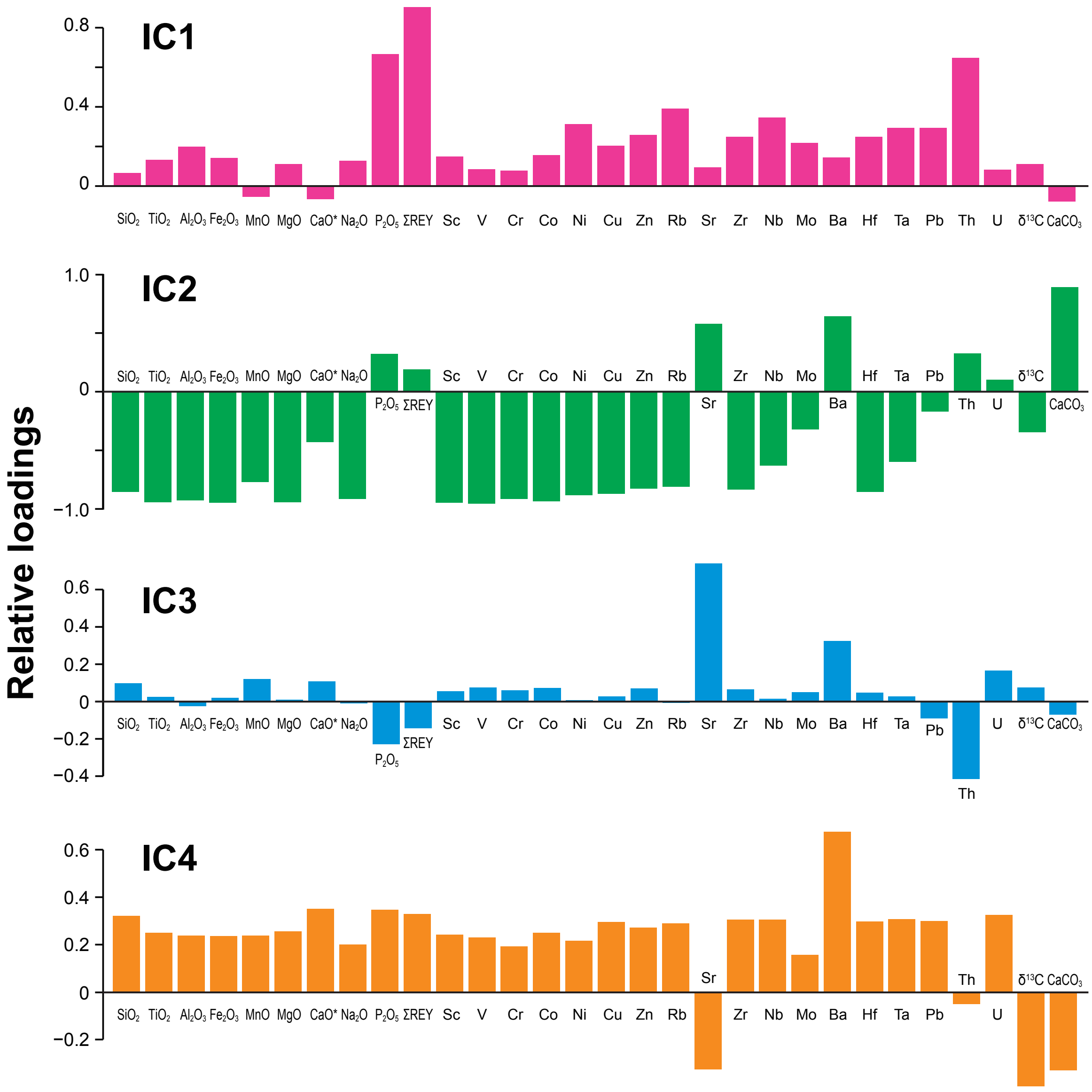

Supplementary Figure S4. Relative loadings of each element for IC1 to IC4 in the result of ICA excluding 'outlier' samples having high IC scores (>3 in absolute value) in the result discussed in the main text.

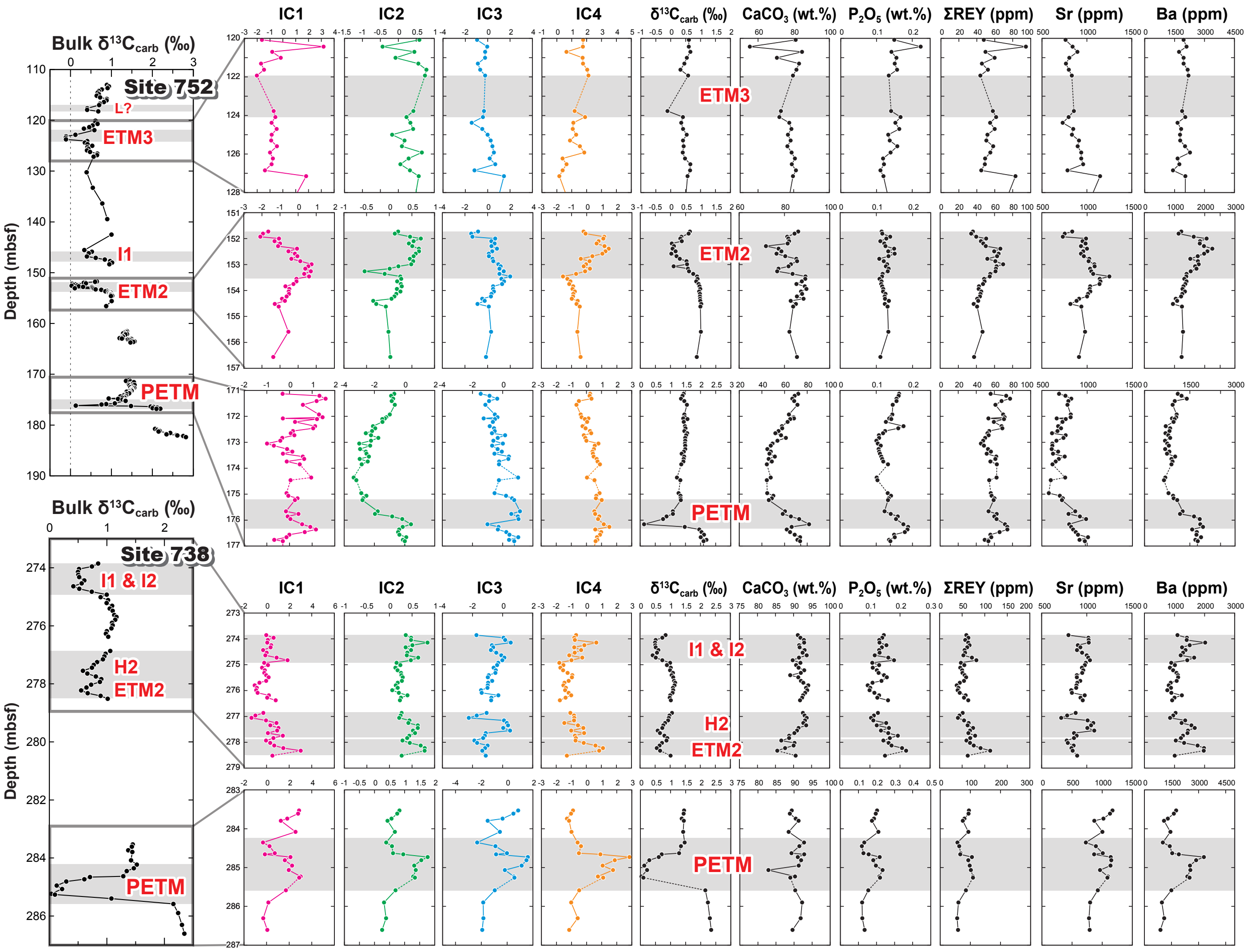

Supplementary Figure S5. Depth profiles of the IC scores in the result of ICA excluding 'outlier' samples having high IC scores (>3 in absolute value) in the result discussed in the main text. The representative geochemical variables are the same as Figure 4 in the main text. Dashed lines indicate the intervals across the horizons of the excluded outlier samples.

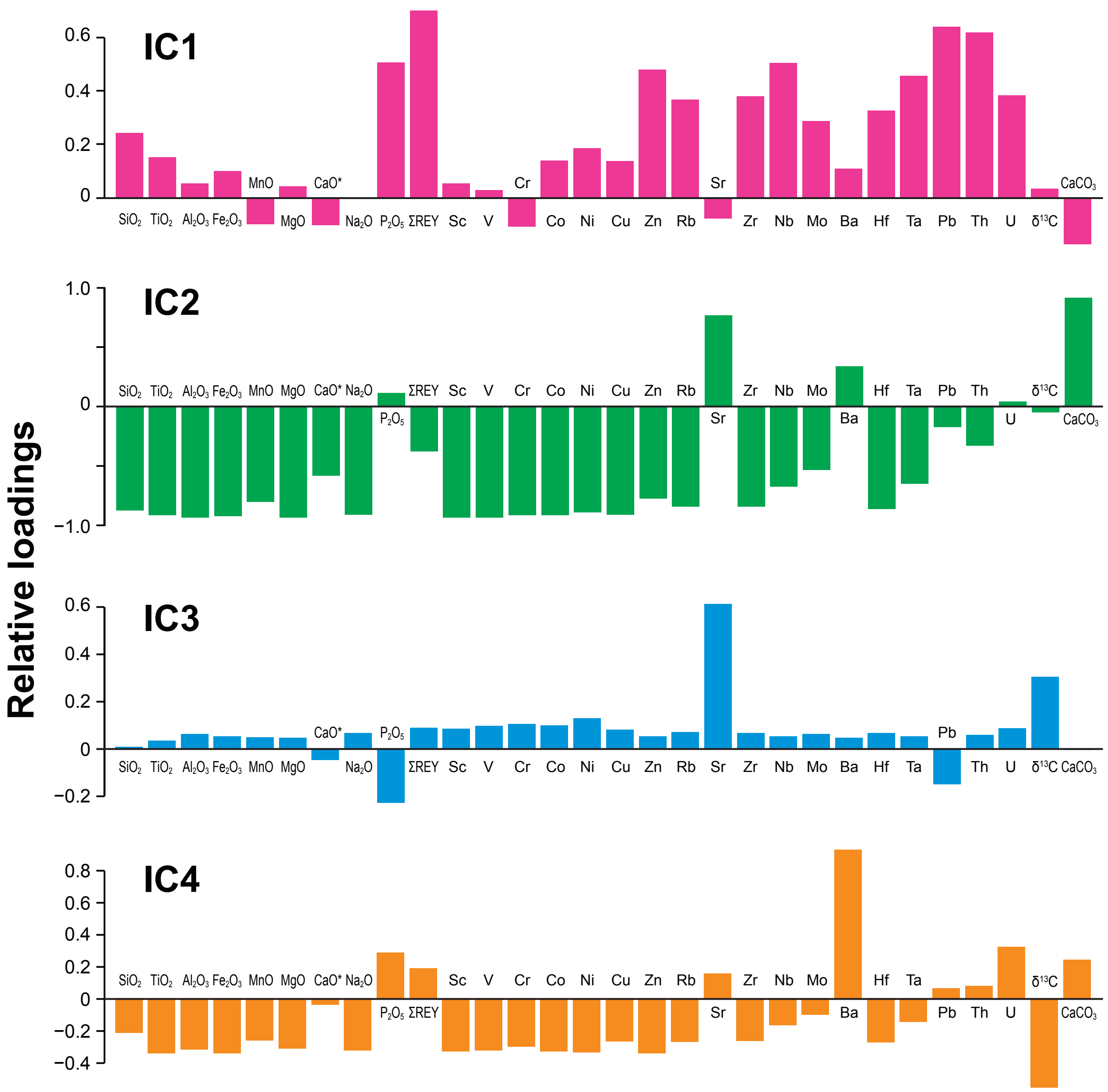

Supplementary Figure S6. Relative loadings of each element for IC1 to IC4 in the result of ICA using the samples from ODP Site 752 only.

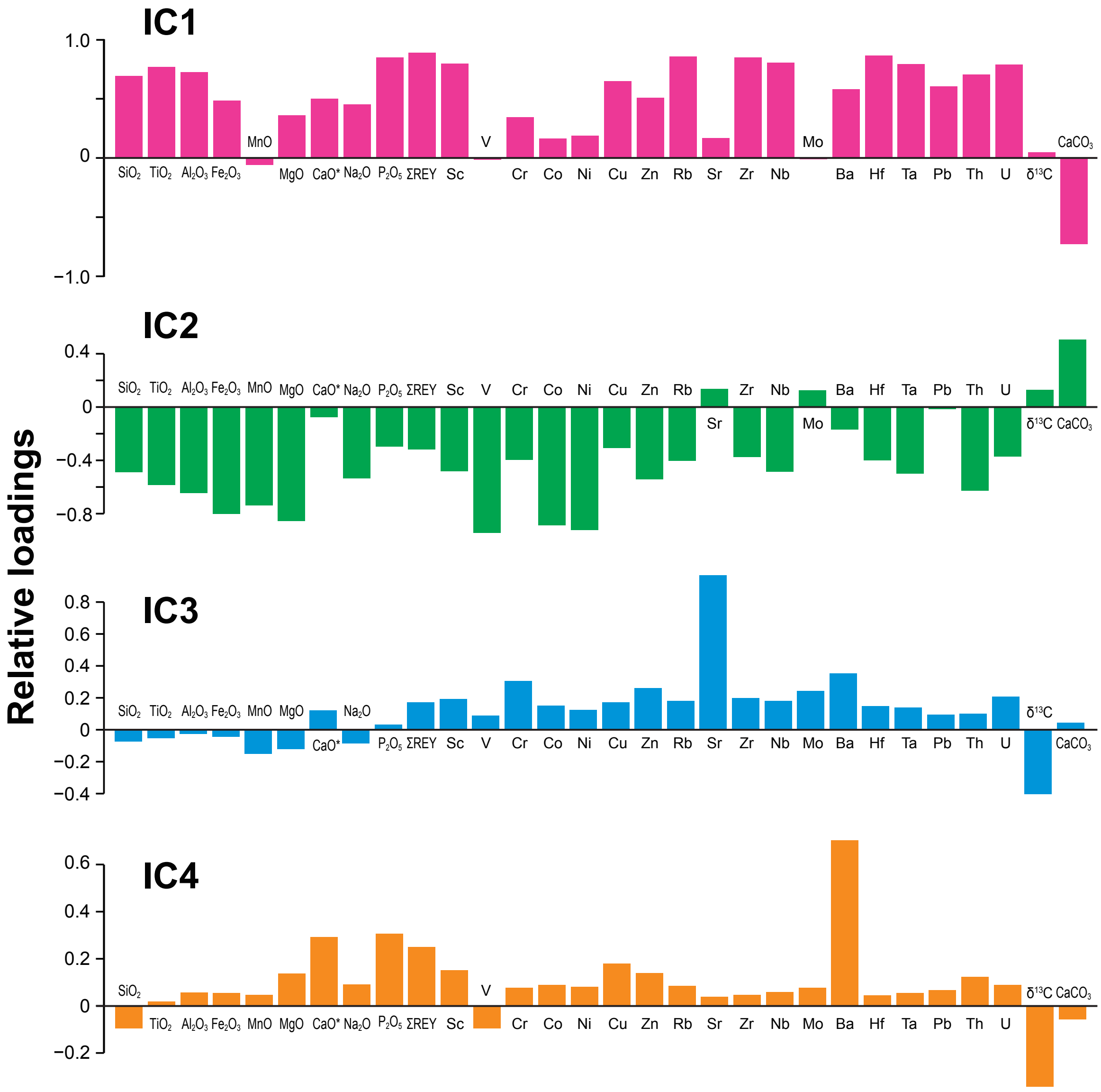

Supplementary Figure S7. Relative loadings of each element for IC1 to IC4 in the result of ICA using the samples from ODP Site 738 only.

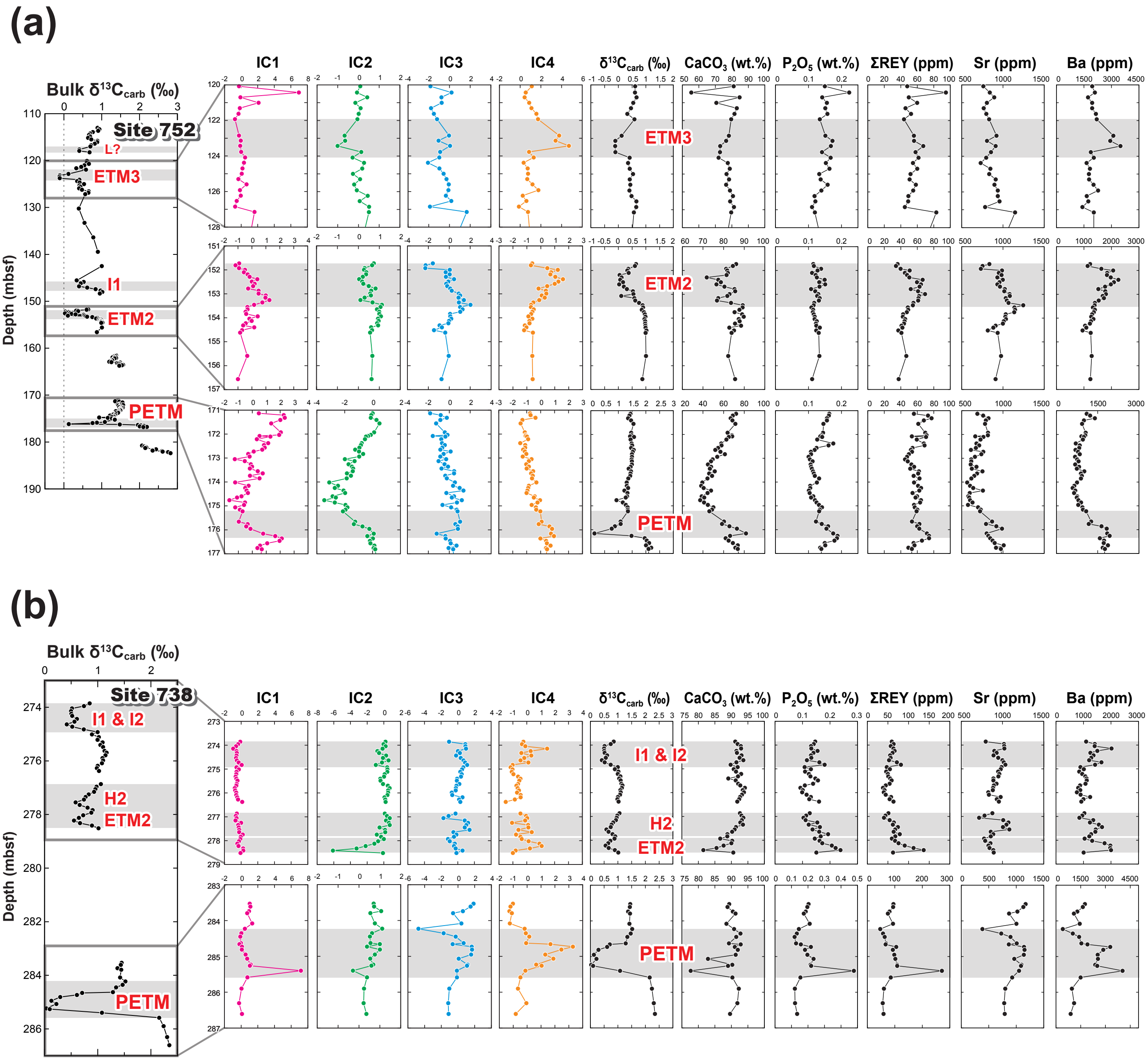

Supplementary Figure S8. Depth profiles of the IC scores in the ICA results using the data from each site separately. (a) ODP Site 752 (n = 173) and (b) ODP Site 738 (n = 76). Note that the ICA results shown in (a) and (b) are independent. The corresponding IC loadings for Sites 752 and 738 are shown in Supplementary Figures S6 and S7, respectively.

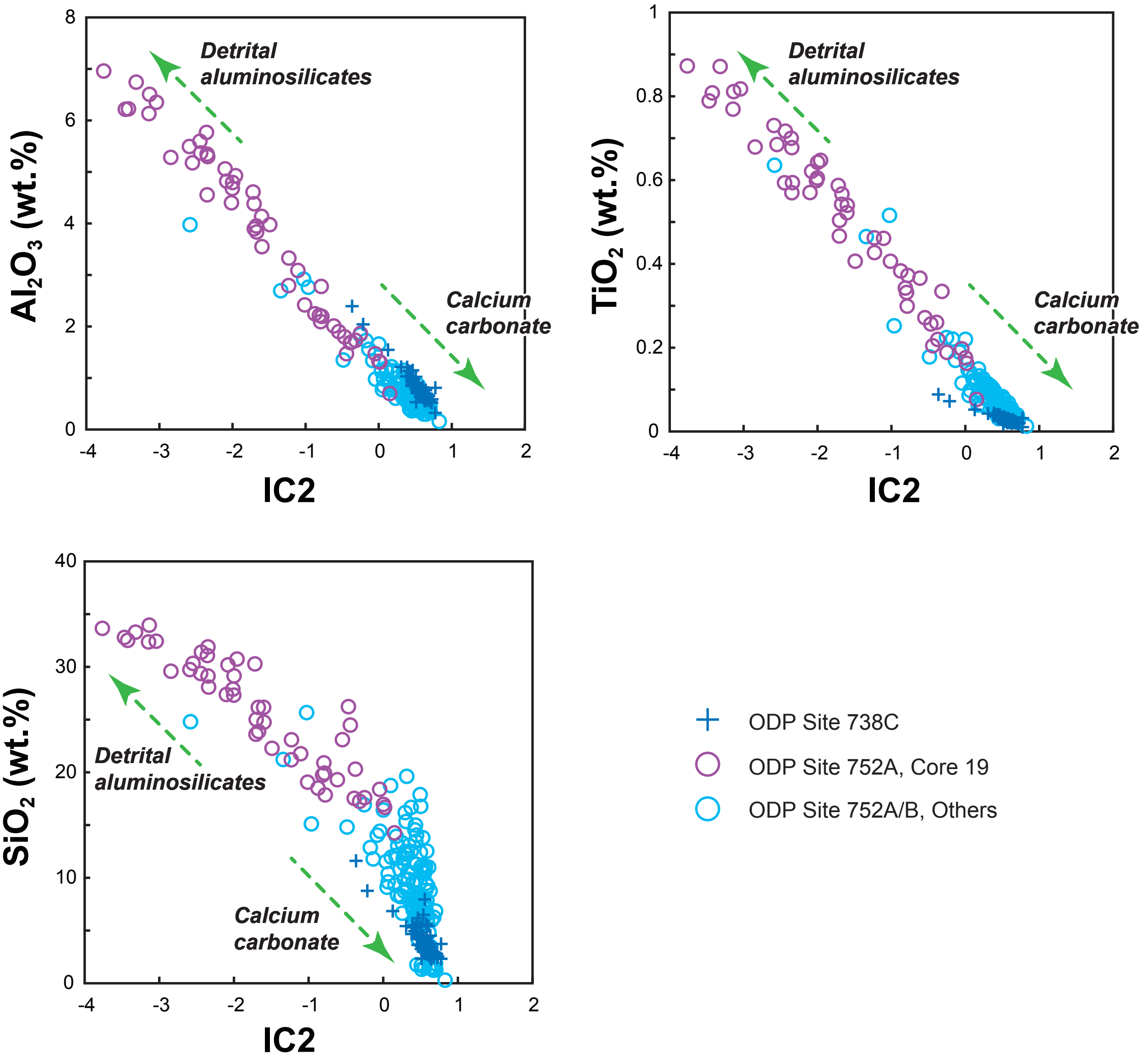

Supplementary Figure S9. IC2 scores versus the elemental contents associated with aluminosilicate materials. (a) IC2–Al_2O_3, (b) IC2–TiO_2, and (c) IC2–SiO_2 diagrams. The samples from Site 752A–Core 19 are highlighted because they are significantly affected by volcanic ash.

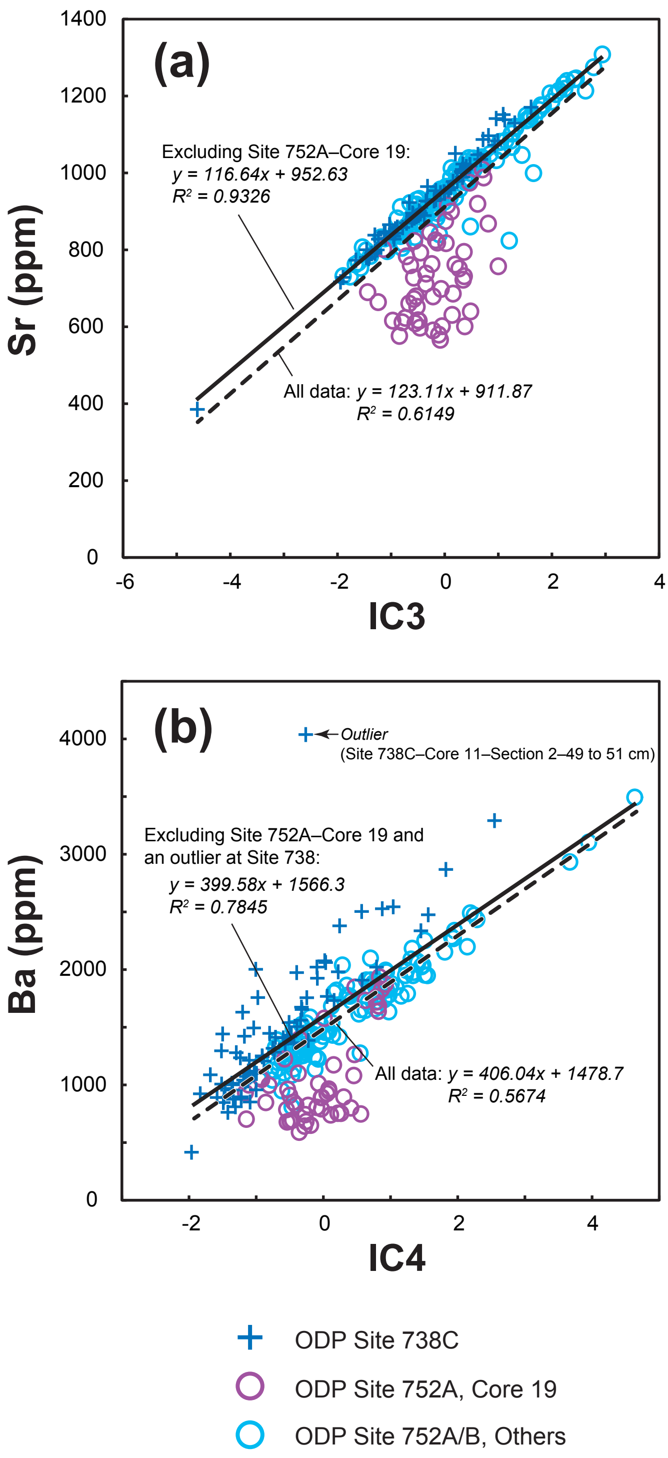

Supplementary Figure S10. IC scores versus elemental contents. (a) IC3–Sr and (b) IC4–Ba diagrams. Linear regressions for all the data are shown in solid lines, and those for data excluding Site 752A–Core 19 and excluding Site 752A–Core 19 and an outlier from the PETM interval at Site 738 in panels (a) and (b), respectively, are shown in dashed lines.

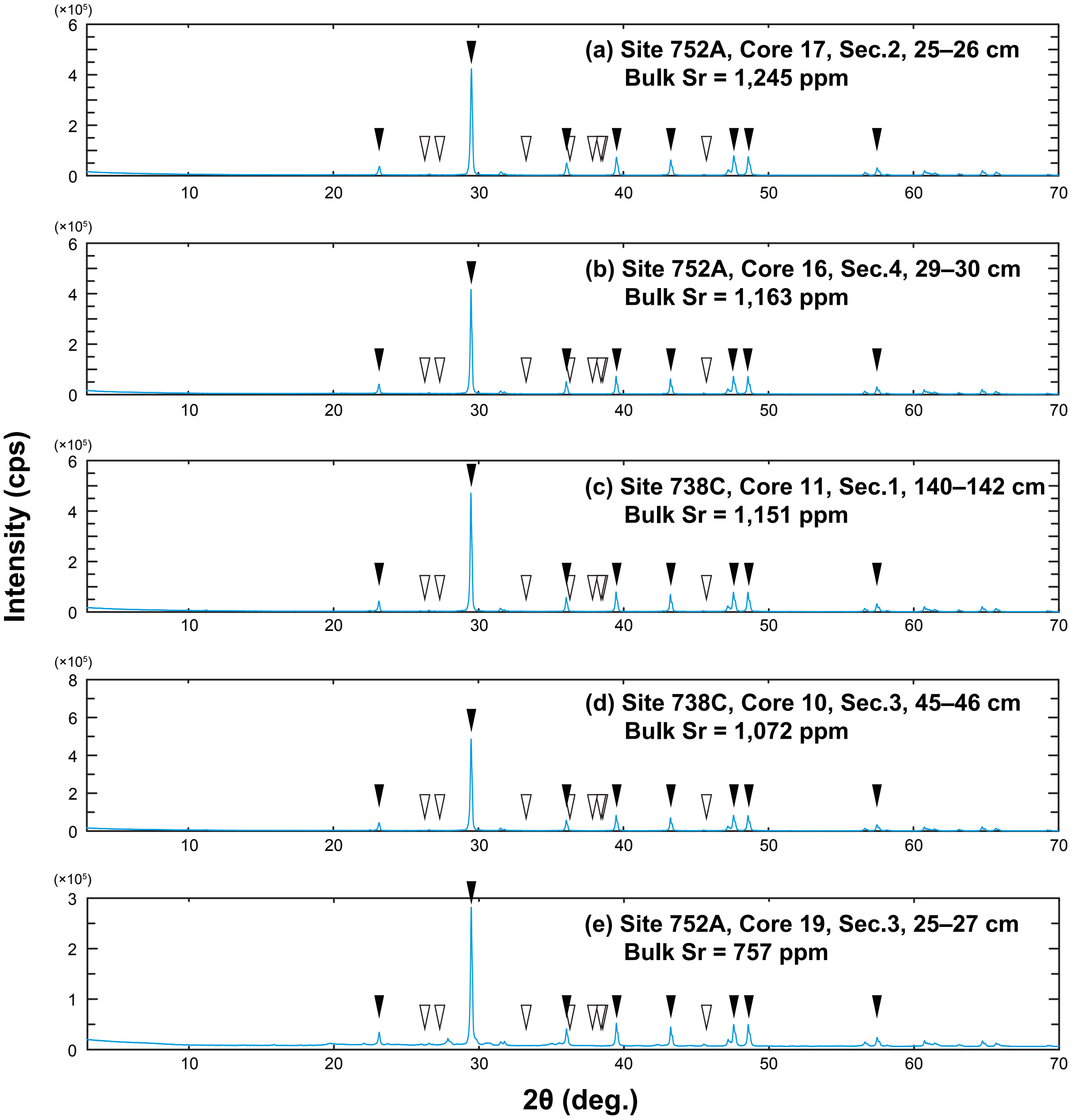

Supplementary Figure S11. X-ray diffractograms for bulk sediment samples from (a) Site 752A, Core 17, Section 2, 25–26 cm, (b) Site 752A, Core 16, Section 4, 29–30 cm, (c) Site 738C, Core 11, Section 1, 140–142 cm, (d) Site 738C, Core 10, Section 3, 45–46 cm, and (e) Site 752A, Core 19, Section 3, 25–27 cm. Black and white triangles indicate the diffraction peaks of calcite and aragonite, respectively. No clear aragonite peak is recognized. Bulk Sr content for each sample is also shown.

Supplementary Table S1. Results of the PCA for the bulk composition and isotopic data from Sites 738 and 752.

	Eigenvalue	Proportion of variance	Cumulative proportion
PC1	19.136	0.6379	0.6379
PC2	3.4037	0.1135	0.7513
PC3	1.5147	0.0505	0.8018
PC4	1.1141	0.0371	0.8390
PC5	0.9859	0.0329	0.8718
PC6	0.8105	0.0270	0.8988
PC7	0.7810	0.0260	0.9249
PC8	0.5373	0.0179	0.9428
PC9	0.3894	0.0130	0.9558
PC10	0.3753	0.0125	0.9683
PC11	0.1872	0.0062	0.9745
PC12	0.1605	0.00535	0.9799
PC13	0.1235	0.0041	0.9840
PC14	0.0979	0.0033	0.9872
PC15	0.0939	0.0031	0.9904
PC16	0.0673	0.0022	0.9926
PC17	0.0537	0.0018	0.9944
PC18	0.0425	0.0014	0.9958
PC19	0.0365	0.0012	0.9970
PC20	0.0282	0.0009	0.9980
PC21	0.0235	0.0008	0.9988
PC22	0.0147	0.0005	0.9992
PC23	0.0067	0.0002	0.9995
PC24	0.0067	0.0002	0.9997
PC25	0.0037	0.0001	0.9998
PC26	0.0023	0.0001	0.9999
PC27	0.0014	0.0001	0.9999
PC28	0.0010	0.0000	1.0000
PC29	0.0006	0.0000	1.0000
PC30	0.0003	0.0000	1.0000

Supplementary Table S2. Estimated loadings for each IC and the standard deviations for each variable in the original data. The loadings correspond to the mixing matrix **A**.

	SiO_2	TiO_2	Al_2O_3	Fe_2O_3	MnO	MgO	CaO*	Na_2O	P_2O_5	ΣREY	Sc	V	Cr	Co	Ni	Cu	Zn	Rb	Sr	Zr	Nb	Mo	Ba	Hf	Ta	Pb	Th	U	$\delta^{13}C$	$CaCO_3$
IC1	-0.53	-0.01	0.05	-0.09	0.00	-0.05	-0.04	-0.02	0.03	20.6	-0.15	-3.86	-2.02	-0.27	1.02	1.10	1.21	0.92	15.0	0.80	0.18	0.01	271	0.02	0.01	0.59	0.40	0.02	-0.05	0.70
IC2	-7.95	-0.21	-1.50	-1.66	-0.02	-0.78	-0.76	-0.36	0.01	-1.4	-5.40	-40.0	-22.0	-6.68	-9.27	-18.1	-17.3	-3.49	95.7	-11.8	-0.68	-0.03	224	-0.30	-0.05	-0.28	0.09	0.00	-0.14	13.7
IC3	-0.19	-0.01	-0.09	-0.06	0.00	-0.04	-0.04	-0.02	-0.01	-0.84	-0.03	0.09	0.01	0.04	-0.07	-0.47	0.35	-0.12	123	-0.02	-0.02	0.00	49.8	0.00	0.00	-0.15	-0.10	0.01	0.12	0.58
IC4	1.41	0.00	-0.04	0.00	0.00	0.02	0.32	-0.01	0.00	0.21	0.09	1.01	0.09	0.13	-0.51	0.55	0.68	-0.01	10.7	0.83	0.05	0.00	406	0.02	0.00	0.13	-0.15	0.03	-0.26	-1.94
Standard deviation of sample data (n = 249)	8.73	0.21	1.53	1.68	0.02	0.80	1.29	0.38	0.04	20.7	5.44	40.5	23.2	6.79	9.70	18.9	19.2	3.81	157	12.7	0.88	0.08	540	0.31	0.06	1.39	0.52	0.12	0.57	14.4

Supplementary Table S3. Relative loadings for each IC. Each value is obtained by dividing original loadings in the Supplementary Table S2 by the standard deviation of the sample data.

	SiO_2	TiO_2	Al_2O_3	Fe_2O_3	MnO	MgO	CaO*	Na_2O	P_2O_5	ΣREY	Sc	V	Cr	Co	Ni	Cu	Zn	Rb	Sr	Zr	Nb	Mo	Ba	Hf	Ta	Pb	Th	U	$\delta^{13}C$	$CaCO_3$
IC1	-0.061	-0.062	0.034	-0.053	-0.159	-0.062	-0.028	-0.054	0.869	0.993	-0.028	-0.095	-0.087	-0.039	0.105	0.058	0.063	0.242	0.095	0.063	0.201	0.098	0.501	0.062	0.171	0.427	0.766	0.208	-0.085	0.049
IC2	-0.911	-0.985	-0.982	-0.987	-0.836	-0.984	-0.584	-0.959	0.132	-0.065	-0.992	-0.987	-0.950	-0.984	-0.956	-0.958	-0.905	-0.915	0.608	-0.931	-0.772	-0.427	0.414	-0.947	-0.740	-0.202	0.178	0.020	-0.245	0.949
IC3	-0.022	-0.037	-0.062	-0.037	0.019	-0.053	-0.030	-0.047	-0.162	-0.040	-0.005	0.002	0.000	0.006	-0.008	-0.025	0.018	-0.030	0.783	-0.002	-0.025	0.042	0.092	-0.013	-0.023	-0.106	-0.192	0.074	0.203	0.040
IC4	0.161	0.017	-0.024	0.001	0.113	0.019	0.249	-0.027	0.086	0.010	0.017	0.025	0.004	0.020	-0.053	0.029	0.035	-0.002	0.068	0.065	0.056	0.034	0.752	0.048	0.076	0.093	-0.285	0.297	-0.450	-0.134
